# Mutated KLF4(K409Q) in meningioma binds STRs and activates *FGF3* gene expression

**DOI:** 10.1016/j.isci.2022.104839

**Published:** 2022-08-03

**Authors:** Alla V. Tsytsykova, Graham Wiley, Chuang Li, Richard C. Pelikan, Lori Garman, Francis A. Acquah, Blaine H.M. Mooers, Erdyni N. Tsitsikov, Ian F. Dunn

**Affiliations:** 1 Department of Neurosurgery, University of Oklahoma Health Sciences Center, Oklahoma City, OK 73104, USA; 2 Clinical Genomics Center, Oklahoma Medical Research Foundation, Oklahoma City, OK 73104, USA; 3 Oklahoma Medical Research Foundation, Genes & Human Disease Research Program, Oklahoma City, OK 73104, USA; 4 Peggy and Charles Stephenson Cancer Center, University of Oklahoma Health Sciences Center, Oklahoma City, OK 73104, USA; 5 Department of Biochemistry and Molecular Biology, University of Oklahoma Health Sciences Center, Oklahoma City, OK 73104, USA

**Keywords:** Molecular biology, Cancer, Transcriptomics

## Abstract

Krüppel-like factor 4 (KLF4) is a transcription factor that has been proven necessary for both induction and maintenance of pluripotency and self-renewal. Whole-genome sequencing defined a unique mutation in KLF4 (KLF4^K409Q^) in human meningiomas. However, the molecular mechanism of this tumor-specific KLF4 mutation is unknown. Using genome-wide high-throughput and focused quantitative transcriptional approaches in human cell lines, primary meningeal cells, and meningioma tumor tissue, we found that a change in the evolutionarily conserved DNA-binding domain of KLF4 alters its DNA recognition preference, resulting in a shift in downstream transcriptional activity. In the KLF4^K409Q^-specific targets, the normally silent fibroblast growth factor 3 (*FGF3*) is activated. We demonstrated a neomorphic function of KLF4^K409Q^ in stimulating *FGF3* transcription through binding to its promoter and in using short tandem repeats (STRs) located within the locus as enhancers.

## Highlights

• ∼15% of meningioma patients share a unique missense mutation K409Q in KLF4
• K409Q mutation occurs in DNA-binding domain and leads to altered DNA specificity
• KLF4^K409Q^ binds to STRs in the *FGF3* gene locus and activates *FGF3* transcription
• FGF3 may contribute to aberrant cell proliferation and meningioma growth

## Graphical abstract

**Figure:**
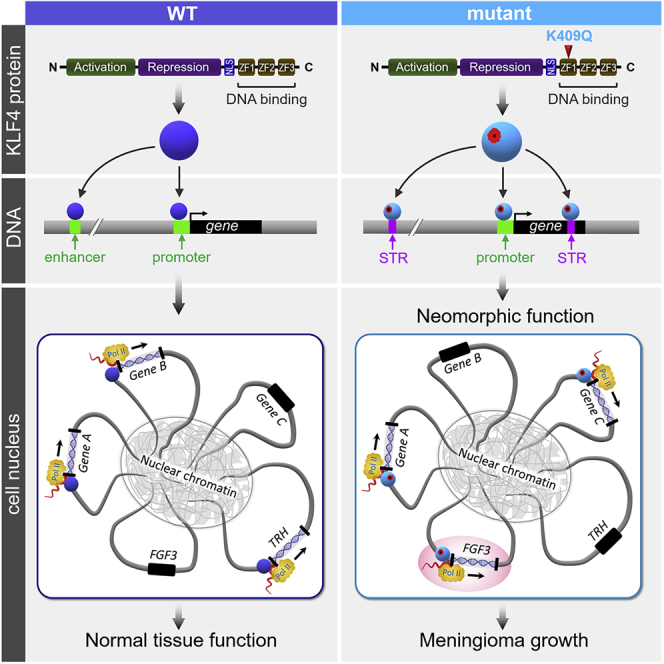

## Introduction

Meningiomas are the most common primary tumor of the central nervous system (CNS) (Ostrom et al., 2013). The genetic landscape of the most common subtype of meningioma involves mutation or copy loss of the *Neurofibromin 2* (*NF2*) gene in approximately 50% of cases (Brastianos et al., 2013; Clark et al., 2013). Other recurrent canonical somatic mutations are present in ∼40% of sporadic meningiomas and are not defined by *NF2* inactivation (Bi et al., 2016, 2017; Clark et al., 2013). These genes include *TRAF7*, *AKT1*, *SMO*, and *KLF4*, among others. Tumors with *KLF4* and *TRAF7* mutations share a unique secretory phenotype (Clark et al., 2013), which is characterized by glandular lumina with secretory globules, and tend to cause disproportional peritumoral edema, which can cause severe medical and neurological complications in pre- and postoperative management (Regelsberger et al., 2009; Reuss et al., 2013). The K409Q mutation in KLF4 is found in ∼15% of meningiomas, and the mutated allele *KLF4*^K409Q^ is the same in all affected patients and occurs together with *TRAF7* missense mutations (Clark et al., 2013). The contribution of the meningioma-specific KLF4 mutation to tumorigenesis and mechanism of action is unknown.

**Figure 1:**
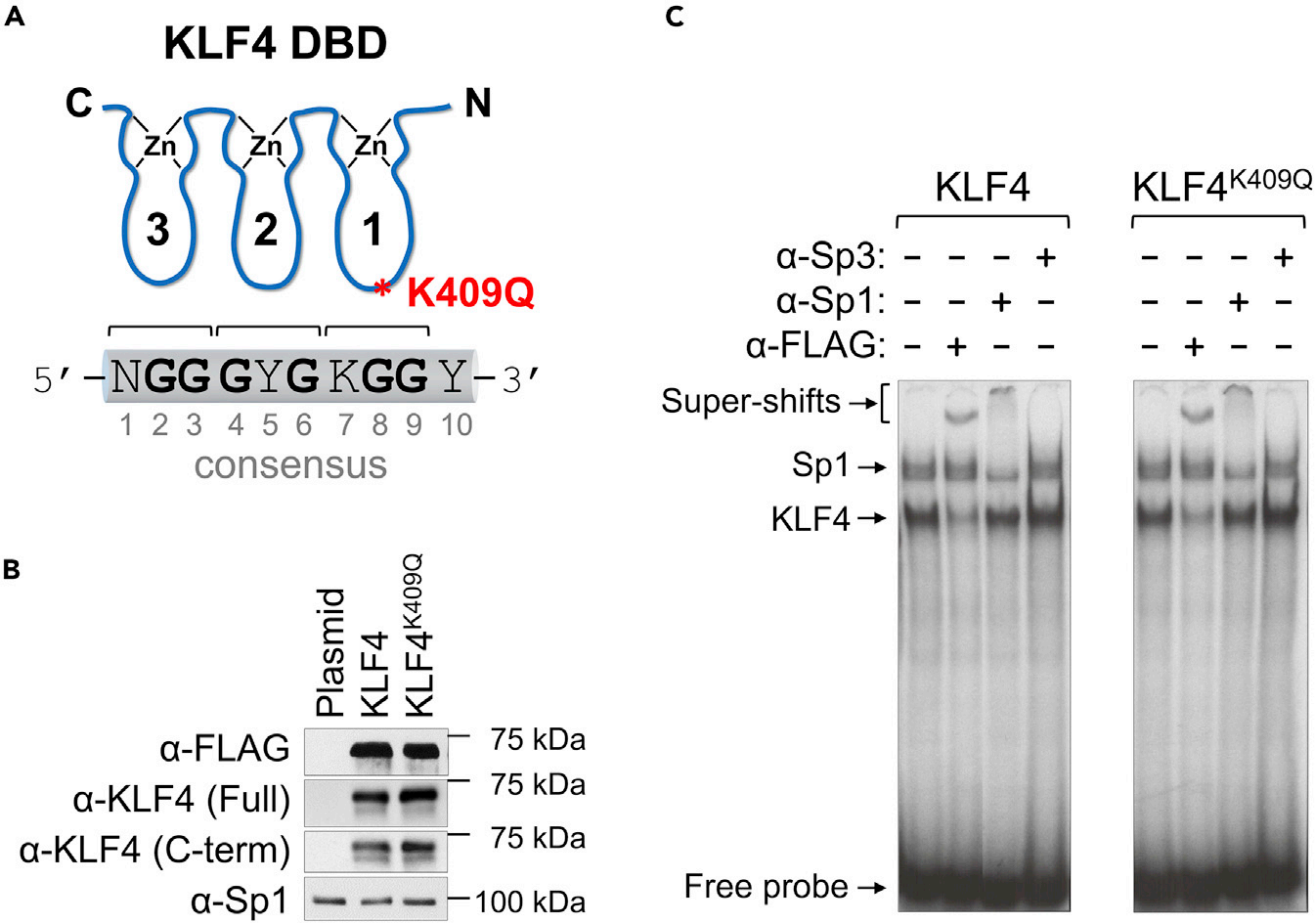
Both WT and mutant KLF4 recognize and bind to the same DNA consensus (A) KLF4 consensus DNA-binding site schematically bound to three protein ZFs. According to conventional recognition code, ZF1 binds nucleotides at positions 7 to 9. K409Q mutation is located within ZF1 (shown as red asterisk). N: any nucleotide; Y: pyrimidine (C or T); K: Keto (G or T). (B) Expression of FLAG-tagged KLF4 proteins in HEK293 cells. Western blot analysis was done using HEK293 whole-cell lysates and indicated antibodies. Anti-KLF4 (Full) and anti-KLF4 (C-term) antibody were raised against full-length recombinant protein and C-terminus peptide, respectively. Anti-Sp1 antibody was used as the loading control. (C) EMSA using nuclear extracts from HEK293 cells transfected with KLF4- or KLF4^K409Q^-expressing plasmids and a 20 bp long oligonucleotide probe spanning KLF4 consensus as described in experimental model and subject details. Arrows indicate specific bands. Antibodies used for super-shifts are shown above the gel.

KLF4 was originally identified as a gut-enriched transcription factor (TF) (Shields et al., 1996); it was shown to be a potent inducer of epithelial cell differentiation and to be one of four transcription factors—along with SOX2, OCT4, and c-Myc—to participate in the reprogramming of adult somatic cells into pluripotent stem cells (iPSCs) (Takahashi et al., 2007; Takahashi and Yamanaka, 2006). It belongs to the Specificity protein (Sp) and KLF (Sp/KLF) TF superfamily (Ghaleb and Yang, 2017), characterized by the presence of three C2H2 zinc fingers (ZFs) within the DNA-binding domain (DBD). The minimal essential binding site for KLF4 was first determined as a DNA heptamer, RRGGYGY (Shields and Yang, 1998). To obtain the crystal structure of all three ZFs bound to DNA, the sequence was extended to a decamer on the 3’-end to accommodate ZF1 (Schuetz et al., 2011). The analysis revealed that the specificity of KLF4 DBD binding to DNA is mediated mostly by ZF2 and ZF3, whereas ZF1 binds outside of the previously established minimal essential binding site and contributes less to specific DNA binding (Schuetz et al., 2011). Nevertheless, the overall structure appears to resemble a conventional “one ZF-three bases” recognition rule (Pabo et al., 2001; Patel et al., 2018), with ZF1 making direct contact with nucleotides at positions 8 and 9 (G8 and G9) within the third triplet of the consensus (Figure 1A).

Without exception, DBDs of SP/KLFs consist of 81 amino acids (aa), suggesting that the fingers act as a single unit with heavy constraints on the DBD structure (Suske et al., 2005). The K409Q mutation resides in the first ZF (ZF1) of KLF4 (Figure 1A). Lysine in this position is conserved in all members of the Sp/KLF superfamily (9 Sp and 17 KLF proteins) (Pei and Grishin, 2015). Importantly, arginine appears in this position in ZF2 and ZF3 of all members of the Sp/KLF family (Kaczynski et al., 2003; Pei and Grishin, 2015; Suske et al., 2005), suggesting essential conservation of a positively charged aa at this position.

In light of this, we hypothesized that the K409Q mutation could trigger an altered DNA specificity of the mutated protein, leading to an alternative gene expression profile, which may in turn contribute to cellular growth potential. In this study, by employing high-throughput RNA-seq analysis, we found KLF4^K409Q^-specific transcriptomes in human cell lines and primary human meningeal cells (HMCs). We discovered *FGF3* as a top gene selectively induced in cells with ectopically expressed KLF4^K409Q^ when compared with wild type (WT). Chromatin immunoprecipitation followed by sequencing (ChIP-seq) demonstrated that KLF4^K409Q^ occupies different binding sites than WT genome-wide and exhibits a preference for an altered recognition motif *in vivo*; moreover, we identified genomic regions bound by KLF4^K409Q^ in the *FGF3* locus. In addition to the minimal *FGF3* promoter, we identified 3 distant regulatory elements, all consisting of short tandem repeats (STRs). These STRs serve as KLF4^K409Q^-dependent enhancers for the *FGF3* promoter, leading to expression of the usually untranscribed *FGF3* gene. We also demonstrated that FGF3 is able to enhance proliferation of a meningioma cell line and its mRNA is detectable in a KLF4^K409Q^-mutated primary tumors. Taken together, our study uncovers a molecular mechanism of how a single, unique K409Q mutation in KLF4 selectively promotes meningeal cell growth in tumors with known *TRAF7* mutations.

## Results

### KLF4^K409Q^ activates *FGF3* expression

To uncover the DNA-binding consequences of the K409Q mutation, we ectopically expressed FLAG-tagged KLF4 or KLF4^K409Q^ in two different cell lines, HEK293 and A549. Both proteins were expressed at similar levels (Figure 1B). We next estimated both WT and mutant KLF4 DNA-binding specificity by electrophoretic mobility shift assays (EMSA), using nuclear extracts from transfected cells and a radiolabeled DNA probe spanning the KLF4 consensus binding sequence (see method details). Anti-FLAG super-shifted the lower band in both panels, suggesting that the lower complex is KLF4 specific (Figure 1C). The addition of anti-Sp1 antibody only super-shifted one of the upper bands, suggesting that this band contains endogenous Sp1. Anti-Sp3 did not super-shift any bands.

**Figure 2:**
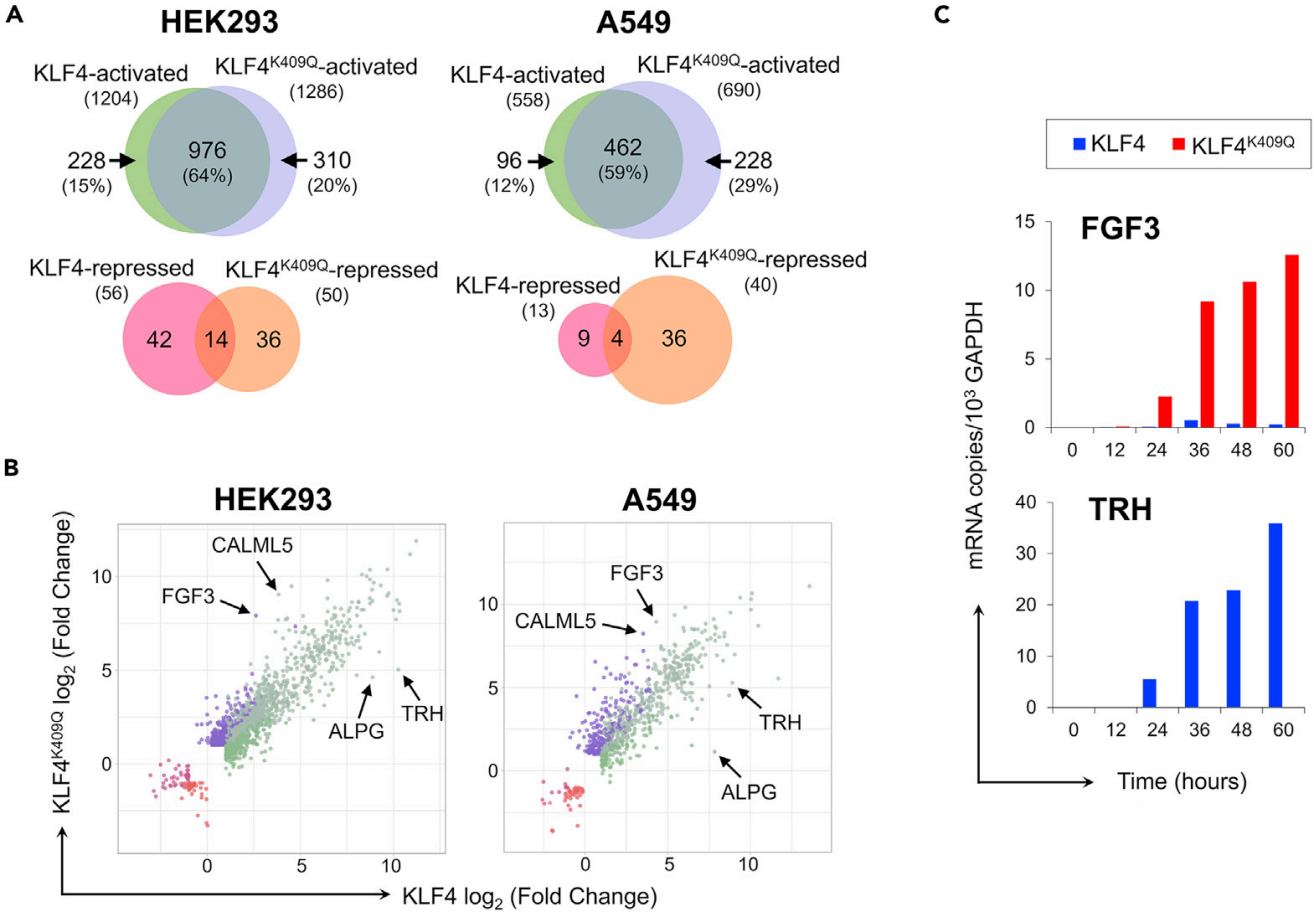
KLF4^K409Q^ activates *FGF3* expression (A) Venn diagram showing the overlap of DEGs in HEK293 or A549 cells by RNA-seq analysis. (B) Scatter-plot of the log_2_ (fold change) of all genes called as significant in KLF4 or KLF4^K409Q^ RNA-seq analysis. Positions of dots corresponding to *FGF3*, *CALML5*, *ALPG*, and *TRH* genes are marked by black arrows. (C) Time course of FGF3 and TRH mRNA expression in HEK293 cells transfected with KLF4- or KLF4^K409Q^-expressing plasmids by RT-qPCR analysis. Number of copies on the *y-axis* is presented as calculated copy number per 1,000 copies of GAPDH mRNA in the same sample (see also Figure S1).

To assess whether altered DNA specificity of KLF4^K409Q^ binding leads to a distinct transcriptional response, we performed RNA-seq analysis following ectopic expression of WT or mutant KLF4 in two different cell lines, HEK293 and A549. We noted 1,606 genes in HEK293 and 835 genes in A549 cells that were either activated or repressed in cells ectopically expressing WT- or mutant-KLF4 compared with untreated cells. The total number of genes activated in HEK293 by either KLF4 or KLF4^K409Q^ was 1,514, whereas the total number of repressed genes was only 92. A similar ratio of activated to repressed genes was found in A549 cells, with 786 activated genes versus 49 repressed genes. These results suggest that both KLF4 proteins function mostly as transcriptional activators. KLF4 upregulated 228 differentially expressed genes (DEGs) in HEK293 cells and 96 DEGs in A549 cells, whereas KLF4^K409Q^ upregulated 310 DEGs in HEK293 cells and 228 DEGs in A549 cells (Figure 2A). The majority of activated genes were shared (64% in HEK293 and 59% in A549), indicating that both KLF4 and KLF4^K409Q^ proteins bound to common regulatory regions in the genome. Interestingly, the list of DEGs activated only by KLF4^K409Q^ is significantly longer in both cell lines (20% and 29% in HEK293 and A549 cell lines, respectively) compared with the list of only KLF4-dependent DEGs (15% and 12%), suggesting that mutant KLF4 has a wider range of gene targets. Direct comparison of the DEG log_2_ (fold change) by KLF4 and KLF4^K409Q^ highlights the shifted transcriptional profile of KLF4^K409Q^ as compared with KLF4, suggesting a potential neomorphic function of the mutant protein. Out of all identified DEGs upregulated by either WT or mutant KLF4, only 36 genes were found to be shared among the cell lines (Figure S1); we inferred that some KLF4^K409Q^-dependent genes may also be upregulated in meningioma cells and promote tumor growth.

Fibroblast growth factor (*FGF3)* and calmodulin-like 5 (*CALML5*) were the most upregulated genes in HEK293 and A549 cell lines with ectopically expressed KLF4^K409Q^ compared with KLF4 (Figure 2B). *FGF3* transcription increased 39- and 25-fold in KLF4^K409Q^-transfected over KLF4-transfected HEK293 and A549 cells, respectively (Figure S1). On the other hand, thyrotropin-releasing hormone (*TRH*) and germ cell type alkaline phosphatase (ALPG) were the most upregulated gene by KLF4 (Figure S1). Detailed analysis of *FGF3* and *TRH* mRNA expression by quantitative PCR (RT-qPCR) showed that *FGF3* mRNA starts to appear in HEK293 cells at 12 h posttransfection with KLF4^K409Q^ and increases dramatically by 60 h (Figure 2C). *TRH* mRNA began to appear at 24 h posttransfection with KLF4 and was still increasing at 60 h. Interestingly, KLF4^K409Q^-transfected cells showed no *TRH* mRNA expression during the entire time course. Thus, our results demonstrated that *FGF3* was most responsive to KLF4^K409Q^, whereas *TRH* was most responsive to KLF4. The latter observation is consistent, with previous studies showing that *TRH* expression is dependent on KLF4 during embryonic hypothalamus development in rats (Guerra-Crespo et al., 2011; Perez-Monter et al., 2011).

### KLF4^K409Q^ binds to and drives transcription from the *FGF3* promoter

**Figure 3:**
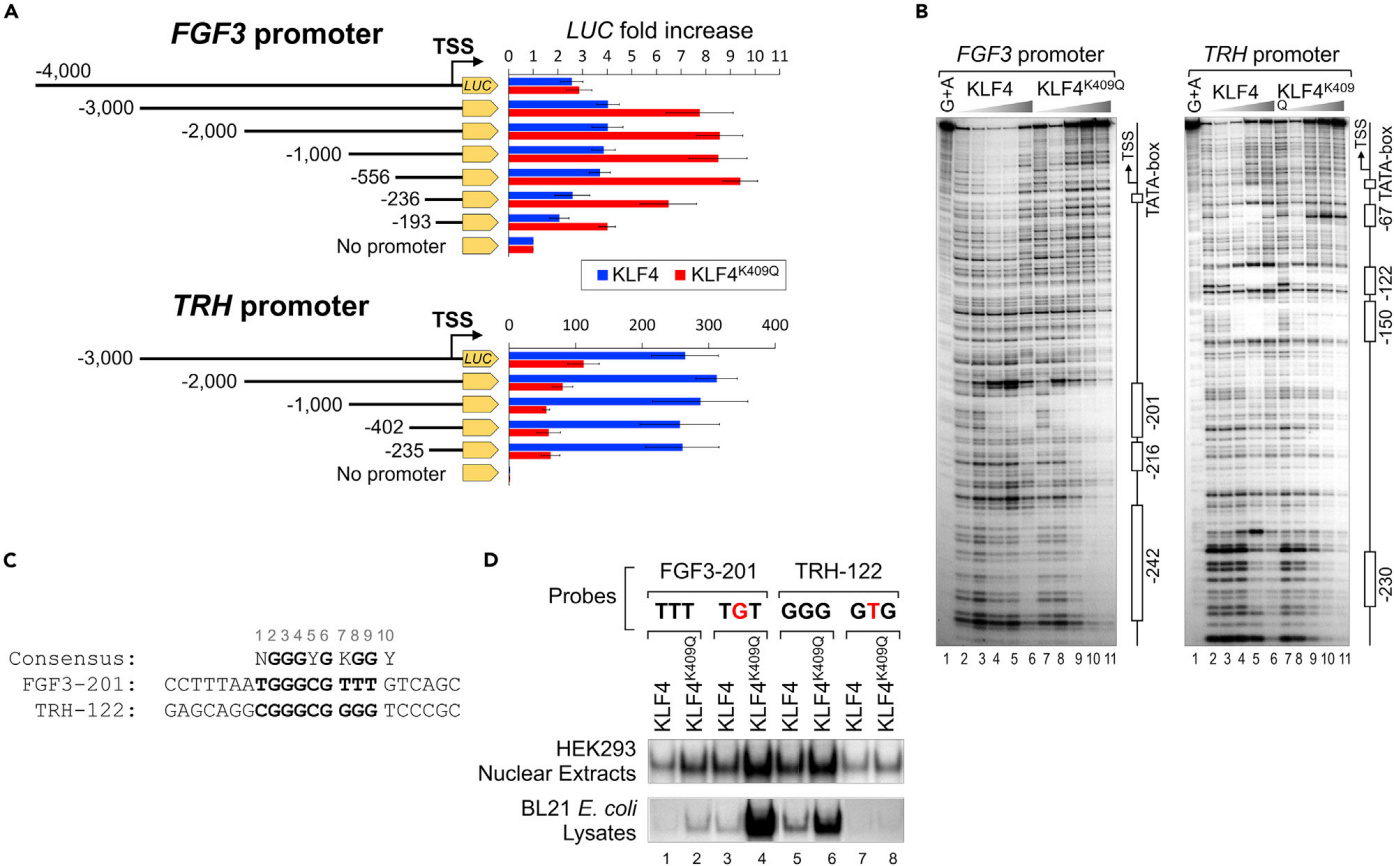
KLF4^K409Q^ binds to and drives transcription from the *FGF3* promoter (A) Activity of luciferase (*LUC*) gene under the control of *FGF3* and *TRH* promoters in HEK293 cells co-transfected with KLF4- or KLF4^K409Q^-expressing plasmids. Luciferase activity was measured 48 h posttransfection. Data are represented as mean ± SD from at least four independent experiments. (B) Quantitative DNase I footprinting analysis of minimal *FGF3* promoter region (−276 to +84 bp) relative to transcription start site (TSS) with increasing amounts of recombinant KLF4 and KLF4^K409Q^ DBD proteins (left panel) and similar analysis of minimal *TRH* promoter region (−270 to +110 bp) (right panel). Positions of protein-binding sites and TATA-boxes are marked by open boxes. Each binding site name reflects the position of the middle nucleotide in the KLF4 site consensus (Y5 in Figure 1A). (C) The alignment of 10 bp KLF4-binding consensus sequence with newly identified strongest binding sites FGF3-201 and TRH-122 in *FGF3* and *TRH* minimal promoters. The 9 bp KLF4-binding sequence in both probes is bolded. ZF1-binding nucleotide triplet is set out by spaces. N: any nucleotide; Y: pyrimidine (C or T); K: Keto (G or T). (D) EMSA analysis of KLF4-binding sites from *FGF3* and *TRH* minimal promoters. Protein/DNA binding was tested in nuclear extracts from HEK293 cells with overexpressed WT or mutated KLF4 proteins (top gel panel) and BL21 *E*. *coli* lysates expressing MBP-KLF4 full-length proteins (bottom gel panel). Sequences of tested probes FGF3-201 and TRH-122 are shown in (C), and their mutants with a single base pair change within the ZF1-binding nucleotide triplet are shown in red. Only protein/DNA complexes are shown (see also Figure S2).

Aligning the human genomic region around the *FGF3* transcription start site (TSS) with a mouse counterpart (Schwartz et al., 2000) revealed that significant sequence homology between two regions only extends to less than 1 kb upstream of the TSS (Figure S2D). We, therefore, tested whether the human *FGF3* promoter contains KLF4^K409Q^-responsive elements. We cloned several *FGF3* promoter fragments truncated at the 5’-end into a luciferase reporter vector. Each promoter fragment contained a TATA-box and ended at position +84 bp relative to the TSS. Each of the constructs was co-transfected with either KLF4- or KLF4^K409Q^-expressing plasmids into HEK293 cells. As shown in Figure 3A, a 3-kb *FGF3* promoter fragment was the longest of the DNA fragments exhibiting significantly higher activity in KLF4^K409Q^-transfected cells than in KLF4-transfected cells. The difference between KLF4^K409Q^- and KLF4-driven activities decreased following promoter truncation down to −193 bp. These findings suggest that a minimal KLF4^K409Q^ responsive element is located between positions −236 and −193 of the *FGF3* promoter. To compare the effect of KLF4^K409Q^ and KLF4 overexpression on *TRH* transcription, we cloned the 5’-end truncated fragments of the *TRH* promoter into luciferase reporter vector. Unlike the *FGF3* promoter, the *TRH* promoter exhibited higher activity in cells transfected with KLF4 than in cells transfected with KLF4^K409Q^ (Figure 3A). These results are consistent with the RNA-seq and RT-qPCR data that showed higher FGF3 mRNA expression in KLF4^K409Q^-transfected cells, whereas TRH mRNA expression was higher in KLF4-transfected cells (Figures 2B and 2C).

To determine whether KLF4^K409Q^ can bind to the *FGF3* promoter, we performed DNase I footprinting (FP) analysis. We used KLF4 DNA-binding domains fused to mannose-binding protein (MBP-KLF4 DBD) purified from BL21 *E*. *coli* (Figure S2A). Increasing amounts of recombinant KLF4 proteins were incubated with [^32^P]-labeled DNA fragments starting from position −276 bp upstream of TSS. After incubation, samples were treated with DNase I, and the digested DNA was examined on denaturing PAGE gel. Figure 3B (left panel) and Figure S2B show that DNA fragments were resolved to the TSS. To prove that generated MBP-KLF4 DBD fusion proteins are acceptable for FP analysis, we compared KLF4 DBD proteins purified from bacteria with commercial recombinant full-length KLF4 protein fused with HIV-1 trans-activator of transcription (TAT), expressed in HEK293 cells. Figure S2B shows that all three KLF4 proteins bound to the *FGF3* promoter displayed similar patterns of DNA protection (lanes 5 to 7 for KLF4; lanes 11 to 13 for KLF4^K409Q^; and lanes 17 to 19 for KLF4-TAT). Detailed analysis of DNA protection by DBD proteins revealed that a site centered at position −201 bp (FGF3-201) was the strongest, as it was protected at the lowest concentration of KLF4^K409Q^ (lane 8 in Figure 3B). In contrast, its protection by WT KLF4 required a higher concentration of the protein (compare lanes 3 to 6 with lanes 8 to 11), indicating a higher affinity of FGF3-201 to KLF4^K409Q^. We also analyzed the upstream part of the *FGF3* promoter between positions −276 bp and −451 bp relative to the TSS and found weak overall protection by KLF4^K409Q^ but not by KLF4 (Figure S2C). Taken together, these results indicate that the *FGF3* minimal promoter favored binding of KLF4^K409Q^ over KLF4.

FP analysis of *TRH* promoter produced the opposite results (Figure 3B, right panel). As expected, KLF4 strongly protected several sites. Among those sites, one at position −122 (TRH-122) displayed the highest protection by WT protein. At the same time, KLF4^K409Q^ demonstrated negligible binding to TRH-122 and nondistinctive overall covering of other regions (compare lanes 3 to 6 with lanes 8 to 11), indicating that the TRH minimal promoter favored KLF4 over KLF4^K409Q^. These observations are consistent with mRNA expression data and reporter gene results, demonstrating that *FGF3* transcription is more responsive to KLF4^K409Q^ and that *TRH* transcription is dependent on KLF4.

### KLF4^K409Q^ binds to an altered motif *in vitro*

Alignment of DNA sequences corresponding to FGF3-201- and TRH-122-protected regions with KLF4 DNA-binding consensus revealed that each region contained a potential KLF4-binding site (Figure 3C). We next examined the binding of KLF4 proteins to FGF3-201 and TRH-122 oligonucleotide probes (Figure 3D). Both KLF4 proteins in HEK293 nuclear extracts displayed weaker binding to FGF3-201 than to TRH-122 (in the upper panel, compare lanes 1 to 5 for KLF4 and 2 to 6 for KLF4^K409Q^). The same was true for proteins expressed in bacteria (lower panel). These findings can be explained by TRH-122’s higher similarity to KLF4 consensus in the third nucleotide triplet and suggest that DNA regulatory elements other than the proximal promoter may also be important for activation of *FGF3* transcription by KLF4^K409Q^. Interestingly, both probes demonstrated stronger binding to KLF4^K409Q^ than to KLF4, despite striking differences in the third triplet sequence (compare lanes 2 to 1 for FGF3-201 and 6 to 5 for TRH-122), suggesting that, paradoxically, the K409Q mutation simultaneously increases affinity and leniency in DNA recognition by KLF4. Next, we substituted a single base pair in the third triplet of each probe: TTT in FGF3-201 was changed to TGT to better match the KLF4-binding consensus, and GGG in TRH-122 was changed to GTG to mismatch the consensus. Mutated probes were tested for binding to KLF4 proteins. As shown in Figure 3D, a mutated FGF3-201 probe displayed an increased binding of KLF4 and KLF4^K409Q^ (compare lanes 1 to 3 and 2 to 4), whereas a mutated TRH-122 probe weakened DNA binding of both proteins (compare lanes 5 to 7 and 6 to 8). These results demonstrated that the consensus G in position 8 is required for DNA binding by both KLF4 proteins and that characteristics that determine DNA-binding specificity of KLF4^K409Q^ are not confined to the third triplet but lie elsewhere.

### KLF4 and KLF4^K409Q^ exhibit distinct binding specificity *in vivo*

**Figure 4:**
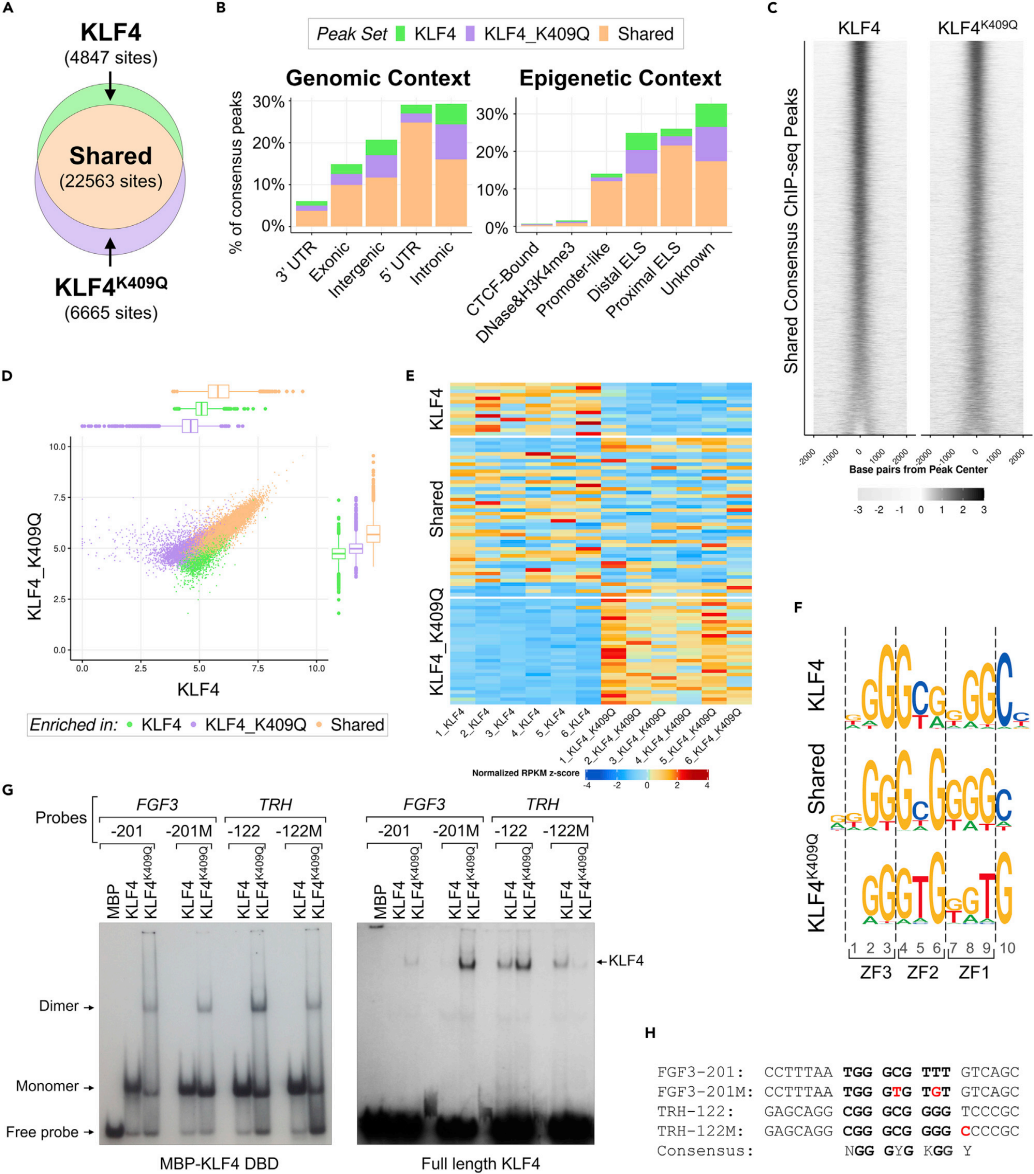
KLF4 and KLF4^K409Q^ exhibit distinct binding specificity *in vitro* and *in vivo* (A) Venn diagram showing the overlap of the peaks in KLF4 and KLF4^K409Q^ sets by ChIP-seq analysis of HEK293 cells with ectopically expressed proteins. (B) Genomic (left) and epigenetic (right) context for the three ChIP-seq peak sets: KLF4, KLF4^K409Q^, and Shared. Categories for epigenetic context are defined by the ENCODE SCREEN project (https://screen.encodeproject.org/). (C) Heatmap of read density of KLF4 and KLF4^K409Q^ ChIP-seq at ranges ±2 kb around consensus peak sets. The read depth was normalized across all six biological replicates for shared consensus peaks. (D) Scatter plot and boxplots of log_2_ average normalized read depth for peaks in each of three consensus sets: KLF4, KLF4^K409Q^, and Shared. (E ) Heatmap of normalized read depth per replicate for the top 10% most variable peaks in each set. Rows represent peaks. Columns represent replicates. (F) Motifs discovered *de novo* from differentially bound sequences within each set in (E). Sequence letter height is correlated with conservation. (G) EMSA analysis using FGF3-201 and TRH-122 sites and their mutants as probes and purified recombinant DBD of MBP-KLF4 or MBP-KLF4^K409Q^ proteins (left panel) or BL21 *E*. *coli* lysates expressing MBP-KLF4 full-length proteins (right panel), as indicated. Free probes and DNA/protein complexes are marked by arrows. (H) Sequence of DNA probes used in EMSA in (G) and mutated nucleotides (in red) are aligned with the KLF4 DNA-binding consensus below. Nucleotides corresponding to the aligned consensus are bolded and nucleotide triplicates binding different ZFs are interspaced. N: any nucleotide; Y: pyrimidine (C or T); K: Keto (G or T). See also Tables S2 and S3.

To investigate whether altered gene expression profiles resulted from different DNA recognition by KLF4 and KLF4^K409Q^ proteins, we transfected HEK293 cells with either KLF4- or KLF4^K409Q^-expressing plasmids and performed ChIP-seq analysis using the anti-FLAG antibody. After performing quality control, we derived a total set of 56,301 unique peaks across all samples meeting a stringent 5% false discovery rate (FDR) threshold (see experimental model and subject details). To reduce false positives, we required that a consensus peak be observed minimally in at least three replicates per group (out of six replicates per group total). This requirement resulted in 27,410 peaks in KLF4-transfected cells, 82% of which overlap with KLF4^K409Q^-binding peaks (Figure 4A). On the other hand, we found 29,228 binding peaks in KLF4^K409Q^-transfected cells, 77% of which overlap with KLF4-binding peaks. The majority of these peaks (22,563 or 66%) were shared by both KLF4 proteins, with 4,847 peaks (14%) specifically occupied in KLF4-transfected cells and the remaining 6,665 (20%) specifically occupied in KLF4^K409Q^-transfected cells.

Next, we examined the genomic context of the peak location and found that shared peaks had a preference to occupy the 5’-untranslated region (UTR) of coding genes (Figure 4B, left chart). Close to half of all KLF4^K409Q^-specific peaks were found in intronic regions in contrast with KLF4. We also characterized the epigenetic context of these peaks by cross-referencing positions of candidate *cis*-regulatory elements (Consortium et al., 2020), which broadly classifies genomic regions into five categories of epigenetic activity (Figure 4B, right chart). Analysis revealed that over half of all peaks were found in genomic regions with active enhancer-like epigenetic signatures (ELS). Approximately 26% (8,878) of peaks were proximal (within 2 kb) to a TSS (proximal ELS), whereas 25% (8,495) of peaks located further away (distal ELS). These data suggest that KLF4 tends to occupy promoter and enhancer regions, whereas KLF4^K409Q^ has similar occupancy profiles with a higher propensity than KLF4 to associate with distal ELS and regions with unknown epigenetic functions.

To further investigate the change in binding genome-wide, we plotted read density heat maps for each dataset. Although many peaks were shared between KLF4 proteins (Figure 4C), the majority (14,826 or 66%) of shared peaks displayed a decrease in KLF4-binding read density in KLF4^K409Q^-transfected cells compared with KLF4-transfected cells. These results suggest that KLF4^K409Q^ binds at sites in the genome that KLF4 normally binds to, and it also binds to additional novel sites. To further examine the differential binding between these factors, we analyzed both peak sets for levels of read enrichment (Figure 4D). A total of 893 unique peaks were found to be differentially bound in KLF4-transfected cells relative to KLF4^K409Q^-transfected cells, highlighting 147/4,847 KLF4-specific peaks, 295/6,665 KLF4^K409Q^-specific peaks, and 451/22,563 shared peaks (Figure 4E).

To determine whether these peaks exhibit differential binding due to sequence variations, we performed specific *de novo* motif analyses on these sets of differentially bound sequences (Figure 4F). Each set demonstrated strong enrichment of motifs containing the accepted KLF4 consensus sequence (Figure 1A). Nevertheless, the analysis revealed significant differences in the DNA-binding sequence preferred by each KLF4 protein. Within the third nucleotide triplet, we found that G9 in KLF4 was replaced by T in KLF4^K409Q^. Interestingly, KLF4^K409Q^ displayed a superior preference for T at position 5, whereas KLF4 favored C over T. Moreover, there was a clear substitution of C10 with G in KLF4^K409Q^-specific motifs. These were unexpected findings because positions 5 and 10 are outside of the canonical ZF1-binding region of KLF4 consensus (Clark et al., 2016; Schuetz et al., 2011) (Figure 1A), suggesting that K409Q mutation induces conformational changes in KLF4 DBD and alters its binding to DNA.

To verify our ChIP-seq findings *in vitro*, we changed FGF3-201 and TRH-122 probes according to newly identified binding motifs and performed EMSA analysis. We substituted C5 to T and T8 to G in FGF3-201M and T10 to C in TRH-122M (Figures 4F and 4H). Two original and two new probes were tested for binding with recombinant DBD and full-length MBP-KLF4 proteins. As anticipated, the FGF3-201M probe displayed stronger binding to KLF4^K409Q^ proteins than did the FGF3-201 probe, whereas KLF4 binding was not affected (Figure 4G), suggesting that the FGF3-201 site is not optimal for KLF4^K409Q^ binding. There may be other regulatory elements in the *FGF3* locus that are important for KLF4^K409Q^-driven gene transcription. On the other hand, the TRH-122M probe had significantly weaker binding to KLF4^K409Q^ but demonstrated the same strong binding to KLF4 protein, indicating that the nucleotide at position 10 is dispensable for WT KLF4 but important for KLF4^K409Q^ DNA recognition.

### KLF4^K409Q^ binding regions in the *FGF3* locus appear to be vast short tandem repeats

**Figure 5:**
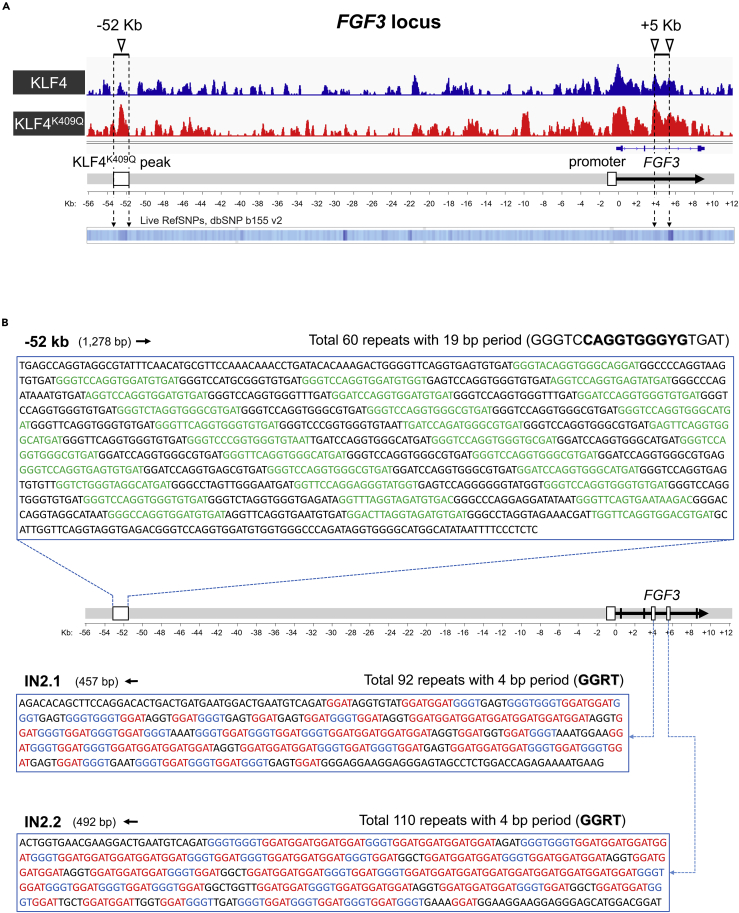
KLF4^K409Q^-binding regions in the *FGF3* locus appear to be vast short tandem repeats (STRs) (A) ChIP-seq analysis of *FGF3* locus. Bigwig tracks display log_2_ ratio of KLF4 ChIP-seq coverage relative to input. One representative replicate for each ChIP-seq condition (KLF4 in blue and KLF4^K409Q^ in red) is labeled on the left side. Six independent biological replicates for each ChIP-seq condition were analyzed and are shown in Figure S3. Schematic position and direction of *FGF3* gene transcription are shown below the tracks. Bottom track shows the *FGF3* locus alignment with heatmap of RefSNPs database. Promoter and STR regions are depicted above the plots (see also Figures S3 and S6). (B) Sequences of *FGF3* locus STRs. Schematic drawing (up-to-scale) of *FGF3* locus shown as a thick grey line. *FGF3* gene and its direction of transcription is shown by black arrow. Exons presented as thin black boxes. Promoter region and STRs are shown as wider open boxes. STR sequences are shown above and below the locus scheme in blow-out windows. Tandem copies of 19 bp repeats in −52 kb STR are labeled by alternating black and green letters. Nucleotides corresponding to the 10 bp KLF4^K409Q^ consensus site are shown above the sequence window with bold letters (Y: pyrimidine, R: purine). Tandem copies of 4 bp repeats in IN2.1 and IN2.2 STRs from *FGF3* intron 2 are marked by red, blue, and black letters. STR lengths (bp) and direction of *FGF3* transcription are marked above each window with number and arrow (see also Figures S4 and S7).

Because KLF4^K409Q^ activates *FGF3* transcription (Figures 2B and 2C) and favors binding to intronic and intergenic regions (Figure 4B), we focused on the *FGF3* genomic locus. This region was discovered on the list of the top 10% of peaks shared by KLF4^K409Q^ and KLF4 (Table S3). Both KLF4 proteins bind sites throughout the entire span of the *FGF3* genomic locus. ChIP-seq signals of KLF4 and KLF4^K409Q^ of a single experiment in HEK293 cells are shown in Figure 5A (see results of all six replicates in Figure S3). One major KLF4^K409Q^-binding peak was found at 52 kb upstream of *FGF3* TSS (FGF3-52 kb region; Figures 5A and 5B). Because the canonical KLF4-binding sequence is G-rich, it is interesting that the coding strand (relative to *FGF3* transcription) of this region contains a high percentage of G and a low percentage of C, resulting in a G/C ratio of 3.4 (Figure S4A). Analysis of the sequence by the Tandem Repeats Finder (TRF) tool (Benson, 1999) revealed that it is an STR consisting of 60 repeats with a 19-bp period (Figures 5B and S4B) (Avvaru et al., 2020; Richard et al., 2008). Further analysis of 19-bp repeats by MEME software (Bailey and Elkan, 1994) revealed the consensus motif in *cis* orientation with *FGF3* transcription. Figures 5B and S4C show that the sequence in the middle of the *FGF3*-52 kb STR motif is very similar to ChIP-seq-derived KLF4^K409Q^-binding consensus at positions 2 to 10 (bottom line in Figure 4F). The only major difference is at position 2: dominant A versus dominant G. However, this position is far outside of the ZF1/DNA contact. More than 50% of the repeats contain T at position 9, which is ideal for binding KLF4^K409Q^. Importantly, the KLF4 consensus sequence (top line in Figure 4F) was not found on either strand of the *FGF3*-52 kb STR. The above results suggest that each of the 60 repeats may potentially bind KLF4^K409Q^ but not KLF4.

The next strongest KLF4-binding region in this locus starts with the *FGF3* promoter and extends across almost the entire length of the gene (Figures 5A and S3). There also are two higher KLF4^K409Q^-specific signals in intron 2 (IN2.1 and IN2.2) located approximately 5 kb downstream of TSS. TRF analysis demonstrated that both fragments are ∼0.5-kb long and consist of 8–24 bp repeats that can be fragmented down to approximately 100 copies of 4 bp core units each (Figures 5B and S4B). Thus, those peaks enclose two very similar STRs designated as low complexity sequences in the human genome. Strikingly, the template strand of both regions exhibits unusually high G content (50% and 53%), whereas C content is very low (3% and 2%; Figure S4A), resulting in G/C ratios of 16.7 and 26.5, respectively. If the most prevalent periodic 4-bp sequences (GGRT) are taken in tandem, they may generate many potential KLF4^K409Q^-binding sites. As a result, a KLF4^K409Q^-specific consensus combination RGGTGGRTG is present 15 and 20 times in IN2.1 and IN2.2, respectively. Interestingly, C nucleotide frequency is very low in the template strand on these STRs, and whatever cytosine is found is not included in the most frequent periodic motifs (Figure S4B) and is located in flanking regions outside of the STR area itself. In sharp contrast, thymine is present at ∼25% rate within the STRs, is always the fourth nucleotide in 4-bp repeats, and is dominant in the KLF4^K409Q^ consensus sequence as T5 and T9 (Figure 4F). On the other hand, C is dominant in the KLF4-binding consensus sequence as C5 and C10 (Figure 4F) and, similarly to the upstream −52 kb STR KLF4 consensus, is not present in either intronic STR.

### *FGF3* locus STRs bind KLF4^K409Q^ and enhance KLF4^K409Q^-specific *FGF3* promoter activity

**Figure 6:**
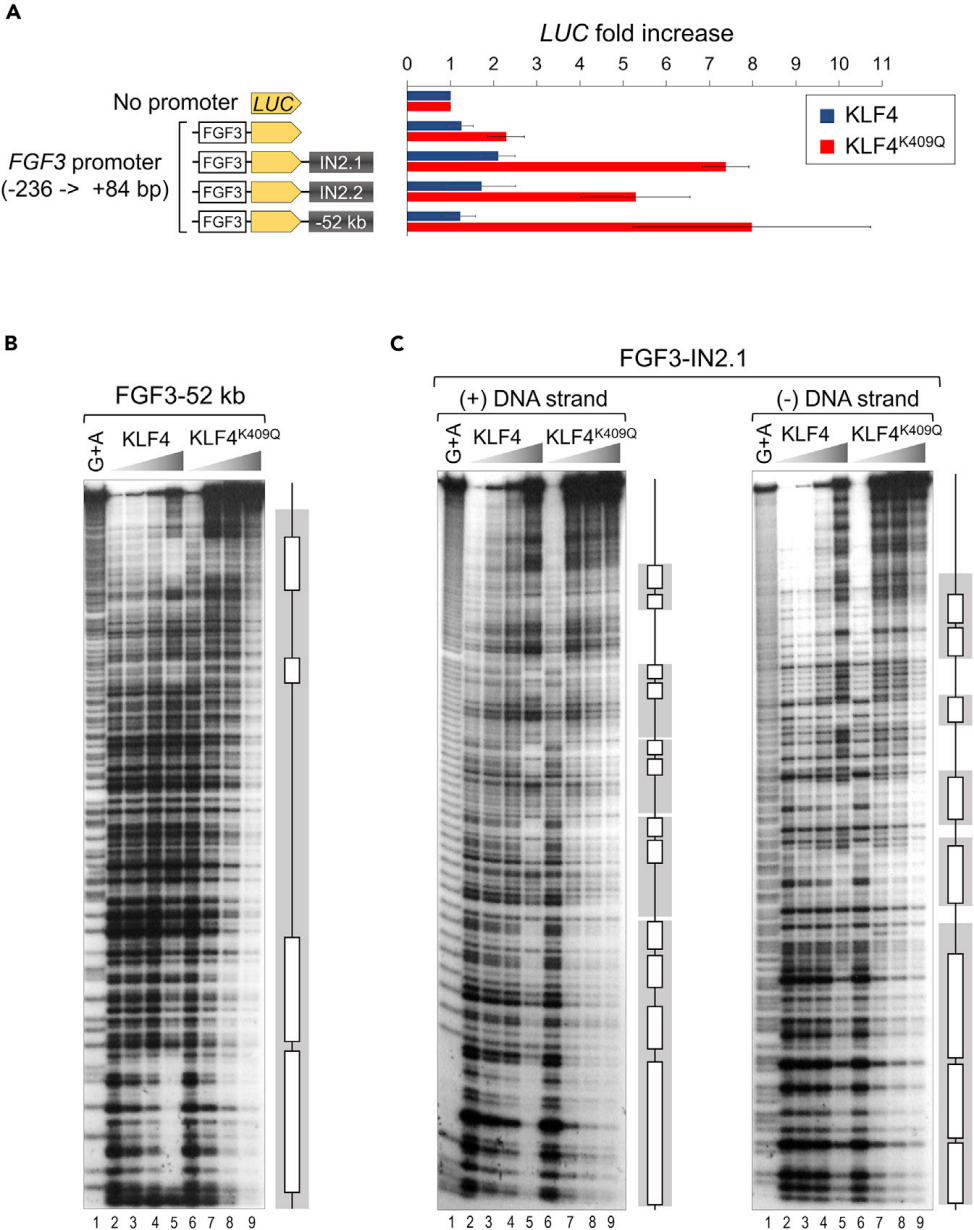
*FGF3* locus STRs bind KLF4^K409Q^ and enhance KLF4^K409Q^-specific *FGF3* promoter activity (A) Activity of luciferase (*LUC*) gene under the control of minimal *FGF3* promoter (from −236 to +84 bp from TSS), alone or with different STRs positioned as enhancers. Reporter vectors were transiently co-transfected with KLF4- or KLF4^K409Q^-expressing plasmids into HEK293 cells. Luciferase activity was measured 48 h posttransfection. Data are represented as mean ± SD from at least four independent experiments. (B) Quantitative DNase I footprinting analysis of the 441 bp DNA fragment from STR-52 kb upstream of *FGF3* TSS (FGF3-52 kb) with increasing amounts of recombinant KLF4 and KLF4^K409Q^ DBD proteins as indicated. G + A ladder is shown as probe sequence marker. Open bars mark areas of KLF4 protein binding. The wide long grey box denotes the binding area of KLF4^K409Q^ protein. (C) Quantitative DNase I footprinting analysis of the 463 bp DNA fragment from STR in *FGF3* intron 2 (FGF3-IN2.1) was performed and marked as in (B). Both coding (+) and template (−) DNA strands were labeled and tested (as indicated) (see also Figure S4).

Next, we tested the ability of *FGF3* STRs to enhance transcription from the minimal *FGF3* promoter. STR fragments (1,278 bp of −52 kb, 457 bp of IN2.1, and 492 bp of IN2.2) were cloned into separate pGL3 reporter vectors immediately after the luciferase gene under the control of minimal *FGF3* promoter (−236 bp to +84 bp relative to TSS) (Figure 6A). The orientation of STRs relative to luciferase gene transcription was kept, as it naturally occurs in the human *FGF3* locus. Next, reporter vectors were co-transfected together with either KLF4- or KLF4^K409Q^-expressing plasmids into HEK293 cells. As shown in Figure 6A, all STRs significantly enhanced KLF4^K409Q^-driven activity of the *FGF3* promoter, while having minimal or almost no effect on KLF4-induced transcription.

We next employed FP analysis to directly investigate the binding of KLF4 proteins to *FGF3* locus STRs. The experiments revealed four specific areas (one strong and three weak) protected by KLF4 in a 441-bp region on the 3’-end of −52-kb STR (lanes 3 to 5 in Figure 6B). In contrast, KLF4^K409Q^ covered the entire DNA stretch without visible clear-cut protected regions (lanes 7 to 9). Due to technical cloning constraints resulting from a repetitive DNA sequence, we were unable to examine the remaining portion of the −52-kb STR. However, one might expect to see the same distinct patterns of binding by KLF4 proteins to the rest of the −52 kb STR, due to its repetitive nature. Because IN2.1 and IN2.2 STRs consist of the same 4-bp repeats, we only examined IN2.1. Because the IN2.1 STR is less than 0.5 kb, FP analysis was done from both directions to encompass the entire length. KLF4 binding was weak, binding several distinct areas and only at the highest concentrations of the proteins (Figure 6C, lanes 5 in both panels). Once again, KLF4^K409Q^ demonstrated strong binding with less distinction between the sites on both strands (lanes 7 to 9). Taken together, these results show that the K409Q mutation in KLF4 makes it prone to bind mini- and microsatellites that contain a set of specific repeats resembling its preferred DNA-binding motif.

### KLF4^K409Q^ activates transcription of *FGF3* in a meningioma cell line and primary human meningeal cells

**Figure 7:**
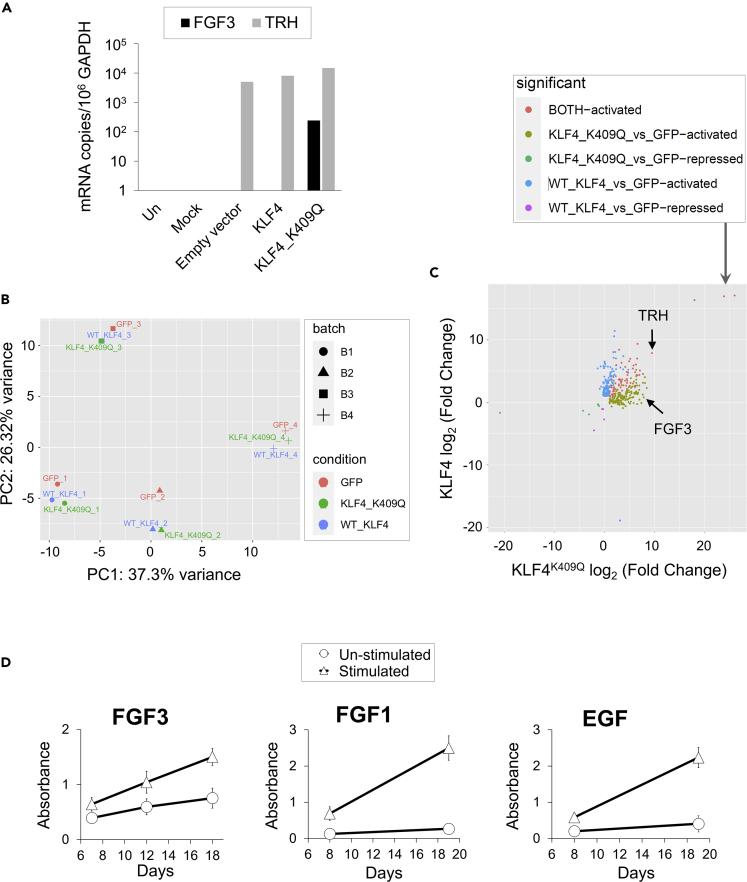
Meningeal cells display KLF4^K409Q^-dependent *FGF3* transcription and proliferate in response to FGF3 (A) *FGF3* and *TRH* mRNA expression in HBL-52 meningioma cells transfected with KLF4- or KLF4^K409Q^-expressing plasmids. Total RNA was purified 48 h posttransfection, and mRNA was amplified by RT-qPCR using GAPDH as internal control. Copy numbers were calculated per 10^6^ copies of GAPDH mRNA in the same sample. (B) PCA Plot of RNA-seq meta-analysis in HMCs transduced with virus expressing KLF4 or KLF4^K409Q^ proteins (https://www.ncbi.nlm.nih.gov/geo/query/acc.cgi?acc=GSE156211. Clustering of samples shows batch effect: instead of clustering by conditions, the samples clustered by their Sample ID (i.e., 1, 2, 3, 4). See also Figure S5A. (C) Scatter plot showing activated and repressed DE genes in meta-analysis of RNA-seq in HMCs. FGF3 and TRH are marked by arrows. See also Venn diagrams in Figure S5B. (D) Proliferation response of meningioma cell line HBL-52 to recombinant FGF3, FGF1, and EGF. Cells were treated with human recombinant FGF3 or FGF1 at 0.1 μg/mL in the presence of 1 μg/mL of heparin or EGF at 5 ng/mL final concentrations in complete medium. Cell proliferation was measured on different days poststimulation (as marked) by Absorbance (OD value at 450 nm) using a Cell Counting Kit-8 [CCK-8] as described in Method details. Assay was set up in 96-well plates (starting at 1,000 cells/well), with eight replicates for each condition and repeated three times. Data are represented as mean ± SD (see also Figure S5, Tables S4 and S5).

After we established that KLF4^K409Q^ could induce expression of *FGF3* in nonmeningioma cell lines, we examined whether it can also activate *FGF3* transcription in a representative meningioma cell line HBL-52. It is an extremely slow-growing grade I meningioma cell line, which harbors no broad copy number alterations but carries a single missense mutation (p.G536S) in *TRAF7* (Mei et al., 2017). Introducing KLF4 with K409Q mutation in this cell line would make it a good experimental model with which to study the role of KLF4^K409Q^ in secretory meningioma development. We transiently transfected HBL-52 cells with KLF4- or KLF4^K409Q^-expressing plasmids and measured *FGF3* and *TRH* mRNA on day 3 posttransfection. As shown in Figure 7A, high levels of *TRH* mRNA were found in all transfected HBL-52 cells, even those transfected with empty plasmid, but *FGF3* mRNA appeared only in cells transfected with KLF4^K409Q^-expressing plasmid, indicating that a meningioma cell line with mutated *TRAF7* and ectopically expressed KLF4^K409Q^ can produce *FGF3* mRNA. These observations are consistent with the results described earlier and show that missense mutations of *TRAF7* and *KLF4* in meningioma cells may lead to *FGF3* expression.

Because the results obtained from using immortalized cell lines might not reflect the actual nature of *FGF3* transcription, we searched the NCBI Gene Expression Omnibus (GEO) database repository for relevant information. We found the expression profiling experiment dataset (GSE156211: “Functional genomics of non-NF2 meningioma development and progression [RNA-seq]”) (A. Sablina, VIB-KU Leuven Center for Cancer Biology in Leuven, Belgium). In this dataset, RNA sequencing was performed to identify DEGs upon transduction of normal human meningeal cells (HMCs), with viral vectors expressing GFP alone or fused with KLF4 and KLF4^K409Q^ proteins. We analyzed raw RNA-seq data files shown in Figure S5A as described earlier for RNA-seq analysis in HEK293 and A549 cell lines. HMCs from four subjects represented four biological replicates for the analysis, as described in (Najm et al., 2021). Principal component analysis (PCA) revealed that clustering of samples showed batch effect, i.e., instead of clustering by conditions, the samples clustered by sample ID (Figure 7B). This batch effect was taken into consideration and corrected in the following DE analysis.

Of 343 DEGs, 141 genes were activated only by KLF4^K409Q^, 116 genes were activated only by KLF4, and both KLF4 and KLF4^K409Q^ activated 74 genes. Seven and five genes were repressed by KLF4^K409Q^ or KLF4, respectively (Figure 7C and Table S4), suggesting once again that both KLF4 and KLF4^K409Q^ mostly function as transcriptional activators in HMCs. In contrast to our observations in HEK293 and A549 cell lines, most activated HMCs DEGs were outside of the overlapping area, which contained only 20% of DEGs. In comparison, KLF4- versus KLF4^K409Q^-specific areas had 37% versus 43% of DEGs, respectively (Figure S5B, left panel and Table S5). Overall, the list of KLF4^K409Q^-dependent DEGs from HMCs and the corresponding list of common 36 DEGs in HEK293 and A549 cells lines share 15 genes (Figure S5C), suggesting that our findings in HEK293 and A549 cells are not restricted to these model cells. These results significantly substantiate the findings obtained in two labs by two different methodologies. Importantly, *FGF3* was the third highest-ranking DEG on the list of KLF4^K409Q^-activated genes in HMCs; it was activated 134-fold (7.07 log_2_ fold change) in KLF4^K409Q^-transduced HMCs over KLF4-transduced HMCs (Table S5 and Figure S5C). It is a higher fold increase than that in HEK293 and A549 cells. On the other hand, TRH was found to be activated by both KLF4 and KLF4^K409Q^ in HMCs (Figures 7C and S5B), which is in contrast to immortalized cell lines. Together with our results in Figure 7A, this finding suggests that *TRH* transcription is less constrained than *FGF3* transcription.

### FGF3 enhances the proliferation of HBL-52 cells and is expressed in KLF4^K409Q^-harboring meningiomas

To examine whether FGF3 increases the proliferation of HBL-52 cells, we treated them with recombinant FGF3 protein in combination with heparin, a cofactor of canonical FGFs (Ornitz and Itoh, 2015). As shown in Figure 7D, FGF3 increased proliferation of HBL-52 cells more than 1.5 times on day 7, steadily increased it more by day 12, and doubled it by day 18, suggesting that KLF4^K409Q^-driven *FGF3* expression may enhance meningioma growth. We used other growth factors, FGF1 and epidermal growth factor (EGF), as positive controls. Figure 7D shows that FGF1 and EGF were more potent in stimulating HBL-52 cell line proliferation than was FGF3.

To demonstrate that FGF3 is expressed in KLF4^K409Q^-harboring meningiomas, we examined primary meningioma tumors available at the OUHSC tissue bank (Table S1). A total of 50 female and male meningioma patients with a median age of 61 years (range 24–89 years) were included in the study. Previous studies have reported that the K409Q mutation of KLF4 always occurs in the context of TRAF*7* missense mutations (Bi et al., 2016). Nineteen patients had *TRAF7* mutations. In addition to mutations in TRAF7, seven patients had co-occurring E17K mutations in AKT1, and six patients had a K409Q mutation in KLF4. RNA purified from these tissues was analyzed by RT-qPCR. Without exception, all meningioma samples express high levels of *KLF4* mRNA isoform 2, varying from 0.1 to 4.4 copies per one copy of *GAPDH* mRNA (Table S1). M−030 *KLF4* mRNA levels were almost equal to *GAPDH* mRNA levels (0.921 copies per 1 copy of *GAPDH*), and M-048 and M-070 *KLF4* mRNA levels were almost twice higher (1.735 and 1.712 copies per 1 copy of *GAPDH*), whereas M-066 and M-068 *KLF4* mRNA levels were even higher (3.9 and 2.5 copies per 1 copy of *GAPDH*). Because our RT-qPCR assay does not distinguish between normal and mutated forms of KLF4, we are not able to estimate the level of the mutant form. Further analysis of *FGF3* mRNA in meningioma tissues revealed that five out of six samples with KLF4^K409Q^ (M-030, M-048, M-066, M-068, and M-070) expressed *FGF3* mRNA (5.2, 21.4, 83.3, 9.8, and 298.4 copies per 10^6^ copies of *GAPDH* mRNA, respectively) (Table S1). Importantly, the rest of the tumor samples, including meningiomas harboring only *TRAF7* mutation or together with mutations in genes other than *KLF4*, had no detectable levels of *FGF3* mRNA. These results demonstrated that only one subtype of meningioma, simultaneously carrying missense mutations in *KLF4* and *TRAF7*, expresses *FGF3* mRNA and strongly suggest that *FGF3* expression is dependent on KLF4 mutation but not TRAF7.

## Discussion

In the present study, we examined a mutation in the transcription factor KLF4 (KLF4^K409Q^) associated with secretory meningiomas. We showed evidence that the K409Q mutation in KLF4 DBD changes its DNA binding and alters transcriptional targets. RNA-seq combined with *in vitro* analyses demonstrated that KLF4^K409Q^ induces *FGF3* transcription. Through ChIP-seq experiments, we found that in addition to *FGF3* minimal promoter, KLF4^K409Q^ binds *FGF3* locus STR regions. Next, we demonstrated how this mutation enables KLF4^K409Q^ to stimulate normally inactive *FGF3* by “hijacking” STRs in *FGF3* locus introns and intergenic region, using them as enhancers for the *FGF3* gene promoter. Furthermore, FGF3 promotes proliferation of human meningioma cell lines. Lastly, we detected *FGF3* mRNA in primary human meningeal cells expressing KLF4^K409Q^ and in human meningioma tumor specimens with a TRAF7/KLF4-mutated genotype. Our findings suggest a neomorphic function of KLF4^K409Q^ is the selective upregulation of FGF3 and that it may promote tumor growth.

Mammalian FGFs regulate many developmental processes, including brain patterning, branching morphogenesis, and limb development. FGF3 belongs to an FGF7 subfamily of paracrine cytokines, which signals through the b splice forms of FGF receptors 1 and 2 (Ornitz et al., 1996). Mice with a homozygous deletion of *FGF3* were viable but showed defects in the inner ear and skeletal development (Mansour et al., 1993). On the other hand, *Bey* heterozygous mutant mice with retroviral insertion in the intragenic region between *FGF3* and *FGF4* developed features that resembled Crouzon-like syndrome of craniofacial dysmorphology (Carlton et al., 1998). Human adults only express *FGF3* mRNA in the cerebellum (GTEx Portal). No other normal tissue has any significant *FGF3* expression. Homozygous mutations in *FGF3* cause hereditary deafness, leading to total inner ear agenesis (Tekin et al., 2007). Recently, *FGF3* was found to be one of the loci associated with craniofacial macrosomia in genome-wide association studies (Zhang et al., 2016). In human malignancies, amplifications of *FGF3* (Gruel et al., 2010) and *FGFR2* (Kunii et al., 2008; Matsumoto et al., 2012; Su et al., 2014; Turner et al., 2010) are linked to breast and gastric cancer. In our studies, no meningiomas had detectable levels of *FGF3* mRNA, except for tumors carrying mutations in TRAF7 and KLF4 (Table S1). Importantly, FGFR2 and FGFR3 were detected in meningiomas (Johnson et al., 2010; Smith et al., 2005; Ueba et al., 1994), and FGF1 was shown to be able to stimulate meningioma cell proliferation by activation of AKT1 and STAT3 (Johnson et al., 2010). Indeed, recombinant FGF3 enhanced proliferation of a meningioma cell line *in vitro* (Figure 7D). These observations indicate that KLF4^K409Q^-dependent *FGF3* expression may lead to meningioma cell proliferation and tumor growth *in vivo*.

Murine *FGF3* gene was initially termed *int-2*, named after its locus, which harbored approximately 50% of mouse mammary tumor virus (MMTV) insertion sites with high incidence of mammary carcinomas (Dickson et al., 1984; Moore et al., 1986). Initially, MMTV provirus insertion sites were mapped in close proximity to 5’ and 3’ ends of the *FGF3* (Casey et al., 1986), leaving the *FGF3* gene uninterrupted for expression (Figure S6). Remarkably, provirus-induced expression of *FGF3*, not just insertion of MMTV provirus, was important for tumorigenesis (Dickson et al., 1984). Moreover, female mice carrying the transgene of *FGF3* cDNA under the control of MMTV LTR sequence developed pronounced mammary gland hyperplasia with a large amount of epithelial tissue in mammary glands, whereas male mice expressing it in the prostate developed benign prostatic hyperplasia (Muller et al., 1990). Interestingly, conditional ectopic overexpression of *FGF3* in the mammary epithelium of transgenic mice showed synergism between the stimulus from estrogen and FGF3 mitogenic pathways, which contributed to the pregnancy-dependent tumorigenesis of FGF3 (Ngan et al., 2002). In addition to the *FGF3* locus (*int-2* in mice), high-throughput analysis of the MMTV insertional mutagenesis identified an activated FGF pathway in 67% of the mammary tumors with virus common insertion sites near *FGFR1* and *FGFR2* (Theodorou et al., 2007). Taken together, these results indicated that FGF3 and its signaling receptors are able to promote tumor growth in epithelial tissues.

Consistent with our observation (Figure 3A), previous analysis of human *FGF3* transcription revealed that a 285-bp promoter fragment upstream of a putative human TSS (Figure S2D) was sufficient to drive the differential expression of the reporter gene in two cell lines (Galdemard et al., 2000). Although different transcription factors, including GATA-4 and SOX family members (Murakami et al., 2004), were shown to be activators of *FGF3* transcription, none of the studies demonstrated a role for KLF4 in this process.

KLFs are transcriptional regulators implicated in a broad range of cellular processes, ranging from apoptosis and proliferation to differentiation, migration, and pluripotency (Limame et al., 2014). The homology of KLF proteins is mainly restricted to DBD, underlying its importance in gene transcription biology. Although most of these functional observations involve differentially expressed normal proteins, the significance of mutated KLFs in cell transformation, including cancer, was established during the last few years. Recurrent mutations of E325 (i.e., E339 in mice) in KLF1 result in severe congenital dyserythropoietic anemia type IV (CDA IV) (Ilsley et al., 2019). In addition to KLF1, two hotspot mutations in *KLF5* were found in colorectal cancer samples: one in DBD and another within a phospho-degron domain (Zhang et al., 2018). In both KLF1 and KLF5, DBD mutations occurred in ZF2, resulting in altered DNA recognition and aberrant transcriptomes. In contrast to these mutations, the K409Q mutation in KLF4, found in a subset of meningiomas, was discovered in ZF1 (Clark et al., 2013). In a recent study, more KLF4 hotspot driver mutations were identified at high prevalence in low-grade intraductal papillary mucinous neoplasms (IPMNs) (Fujikura et al., 2020). In addition to K409Q, three other missense mutations (K409E, S411Y, and S411F) were recently detected in ZF1 of KLF4. The authors suggested that exclusive association of these mutations with low-grade IPMNs highlights distinct molecular features of low-grade and high-grade dysplasia. Similarly, KLF4^K409Q^ was found only in low-grade meningiomas (Clark et al., 2013). Because these mutations are close to each other, they may change the mutated KLF4 protein structure and function similarly. Thus, it may be of interest to determine how IPMN mutations would alter DNA recognition by KLF4 and whether those changes will be similar to KLF4^K409Q^-induced alteration.

The DNA-binding specificities of almost all members of the KLF family were determined by using high-throughput ChIP sequencing (Burdach et al., 2014; Ilsley et al., 2019; Knoedler et al., 2017; Wang et al., 2018; Zhang et al., 2018) and/or SELEX (Fornes et al., 2020; Jolma et al., 2013). The consensus motif for the whole family can be summarized as NGGG**Y**G**K**GGY (Figure 1A), underpinning the general rule that transcription factors related in amino acid sequences generally bind to similar sites (Jolma et al., 2013). It is interesting that the recognition sequences obtained by ChIP-seq for mutated KLF1 and KLF5, where glutamic acid in the same position of ZF2 was replaced with lysine, also recognized somewhat similar motifs. KLF1^E339K^ was found to bind RGGG**R**G**G**GGN (Ilsley et al., 2019), whereas KLF5^E419K^ bound NGGG**G**G**Y**GGN (Zhang et al., 2018). Curiously, KLF5^E419K^ strongly prefers G in position 5, whereas KLF1^E339K^ prefers G in position 7, which is outside of the ZF2 direct contact area. These results suggest that one amino acid substitution within ZF2 defines DNA recognition by the whole DBD. Likewise, in our ChIP-seq experiments, DNA recognition motifs derived for KLF4 and KLF4^K409Q^ show differences at positions 5, 9, and 10. KLF4^K409Q^ prefers T5, T9, and G10, whereas KLF4 prefers C5, G9, and C10 (Figure 4F). Taken together, these results provide additional evidence that structure-function dynamics of ZF/DNA interaction can cause deviation from the conventional “one ZF-three base pairs” protein/DNA recognition pattern (Patel et al., 2018).

STRs take up more than 2% of the human genome (Toth et al., 2000). In the present study, we identified one STR located 52 kb upstream of the *FGF3* gene and two other STRs in *FGF3* intron 2 (IN2.1 and IN2.2). Each of these enhances *FGF3* promoter-driven reporter transcription and consists of multiple tandem KLF4^K409Q^-binding sites. *FGF3*-52 kb STR is classified as a minisatellite (Liehr, 2021) and is unique in the human genome; this is a class of repetitive elements with a unit length of 10–60 bp and a conserved core that spans 0.1–15 kb (Avvaru et al., 2020; Richard et al., 2008). Analysis of the mouse genome found several similar minisatellites in other regions/chromosomes but not in the *FGF3* locus. It consists of 60 copies of 19 bp tandem repeat with a conserved principal sequence (Figure S4C). Each copy carries a potential binding site for KLF4^K409Q^. Thus, the −52 kb STR region is potentially able to bind up to 60 molecules of mutant KLF4 and constitutes an upstream KLF4^K409Q^-specific enhancer for *FGF3* gene transcription.

*FGF3*-IN2.1 and IN2.2 can be designated as microsatellites, varying (1–6 bp) length stretches of repetitive DNA motifs (Liehr, 2021). They consist of GGAT and GGGT tetra-nucleotides, which in tandem order form a KLF4^K409Q^ consensus motif (Figure 4F) in *trans* orientation with *FGF3* gene transcription. The abundance of similar repeats in the human genome provides material for evolution, but it comes at the expense of genomic instability that in many cases leads to repeat expansion diseases (REDs). Expansion of certain tri-nucleotide-based repeats of varying lengths in exon or noncoding regions affects the nervous system, resulting in neurodegenerative disorders such as Huntington disease, fragile X syndrome, and other nucleotide repeat expansion disorders (reviewed in Khristich and Mirkin, 2020; Ramakrishnan and Gupta, 2021; and Rodriguez and Todd, 2019). Limited experimental information is available about the exact function of microsatellites located in noncoding regions of the genome. Their role is mostly tied to chromatin compartmentalization and genome packaging (Kumar et al., 2010). For example, GAA repeat expansion was shown to lead to epigenetic changes in the genome (Al-Mahdawi et al., 2008), and the AAGAG repeat transcribed RNA was an essential component of the nuclear matrix (Pathak et al., 2013). Although there are no REDs linked to *FGF3* STRs described here, the only known disease caused by the expansion of intronic (CCTG·CAGG) tetra-nucleotide repeats is myotonic dystrophy 2 (DM2), one of the two most common forms of muscular dystrophy (Koscianska et al., 2021). Interestingly, the expansion of these repeats in DM2, bidirectional transcription, and repeat-associated non-ATG (RAN) translation leads to pathological expression of poly-(LPAC) protein in *cis* orientation and poly-(QAGR) protein in *trans* orientation (Zu et al., 2017).

The proliferation of high-throughput sequencing data revealed that only 1%–2% of the transcriptome encodes functional proteins, whereas the rest gives rise to an abundance of noncoding RNAs, including transfer, ribosomal, micro, and other RNAs. A recent addition to the expanding list of regulatory RNAs is the emerging class of enhancer RNAs (eRNAs). eRNA production is a widespread phenomenon implicated in gene expression regulation in multiple cell types in response to various stimuli (Sartorelli and Lauberth, 2020). Thus, there is a plausible possibility of eRNA production from both IN2.1 and IN2.2 STRs in cells expressing KLF4^K409Q^. In this situation, *trans* eRNA transcription from IN2.1 and/or IN2.2 STRs in *FGF3* gene, if translated (Goujon et al., 2010, Heger and Holm, 2000), may lead to the expression of a number of poly-(GWV/MD) peptides (Figure S7). On the other hand, *cis* eRNA transcription from both STRs, if translated, will result in the expression of poly-(PSI/TH) repetitive proteins up to 164 aa long (Figure S7). The experiments addressing the possibility of *FGF3* STR eRNA existence and the function of the resulting proteins are outside the scope of this study.

In summary, these findings demonstrate that a single-point mutation (K409Q) in transcription factor KLF4 can confer a neomorphic “gain of function.” Our *in vitro* binding results show a definitive molecular mechanism of how this mutation leads to altered DNA-binding specificities, resulting in altered transcriptional targets and growth-promoting properties that may underlie its specific phenotype. This study provides the foundation for understanding the biological features of secretory meningioma and possibly other tumors, which may help identify putative therapeutic targets.

### Limitations of the study

In this study, we prosecuted *FGF3*, the top KLF4^K409Q^-upregulated gene, and demonstrated its ability to contribute to meningioma growth. The relative impact of FGF3 on secretory meningiomas with KLF4 mutation remains unclear and deserves further study supported by a larger number of primary tumor samples. Examination of the role of KLF4^K409Q^ in *FGF3* activation by direct gene editing in meningeal cells would be challenging due to the extremely slow growth and limited lifespan of these cells in culture. Because a KLF4^K409Q^ mutation occurs exclusively in meningiomas harboring a missense TRAF7 mutation, the significance of *FGF3* expression should be studied in the context of TRAF7 neomorphic function. However, little is known about the role of mutated TRAF7 in meningioma development. Thus, the roles of both mutant KLF4 and TRAF7 in growth and the secretory clinical manifestation of meningiomas also merit further analyses. We found at least 14 other candidate genes upregulated specifically by KLF4^K409Q^ (Figure S5C), which may be responsible for tumor growth and its secretory phenotype. The analysis of the comprehensive molecular mechanism of each of those genes in secretory meningioma development is a matter for future studies and outside the scope of the present paper.

## STAR★Methods

### Key resources table

.

**Table:** 

REAGENT or RESOURCE	SOURCE	IDENTIFIER
**Antibodies**		

Monoclonal ANTI-FLAG® M2-Peroxidase (HRP) antibody produced in mouse	Sigma-Aldrich	Cat#A8592; RRID: AB_439702
Anti-KLF4 antibody produced in rabbit (HIS-tag recombinant hKLF4)	Sigma-Aldrich	Cat#SAB1300678; RRID: AB_10604417
Anti-Sp1 antibody, rabbit polyclonal	Sigma-Aldrich	Cat#07–645
Anti-SP3, C-Terminal antibody produced in rabbit	Sigma-Aldrich	Cat#SAB4502839; RRID: AB_10744669
Anti-KLF4 antibody, rabbit polyclonal (against c-terminus region including K409)	Sigma-Aldrich	Cat#SAB2107958

**Bacterial and virus strains**		

One Shot™ BL21(DE3) Chemically Competent *E*. *coli*	Fisher Scientific	Cat#C600003

**Biological samples**		

Meningioma primary tumor tissue	OUHSC tissue bank (Neurosurgery)	Biospecimen & Tissue Pathology | Stephenson Cancer Center - OU Health

**Chemicals, peptides, and recombinant proteins**		

MBP-KLF4 DBD	This paper	N/A
MBP-KLF4^K409Q^ DBD	This paper	N/A
Recombinant Human KLF4-TAT	Sigma-Aldrich	Cat#SRP3101
Recombinant Human FGF3	R&D Systems	Cat#1206-F3-025
Recombinant Human FGF1	R&D Systems	Cat#232-FA-025
Recombinant Human EGF	R&D Systems	Cat#236-EG-200
Heparin Solution	StemCell Technologies	Cat#07980

**Critical commercial assays**		

3′ end Tag RNA Sequencing, Library Preparation	Lexogen	Cat#015.96
NextSeq HO SR75, Nextseq	Illumina	Cat#20024906
DNA Library Preparation (for HEK293 ChIP-seq)	Swift Bioscience	Cat#21096
NovaSeq 400M-1599M reads	Ilumina	Cat#20028312
Direct-zol™ RNA MiniPrep Plus	Zymo Research Corporation	Cat#R2071
RNeasy Mini Plus Kit	QIAGEN	Cat#74136
QIAshredder	QIAGEN	Cat#79656
RNAlater® Solution	Fisher Scientific	Cat#AM7023
Lipofectamine™ LTX Reagent	Invitrogen	Cat#15338100
Lipofectamine™ 3000 Transfection Reagent	Invitrogen	Cat#L3000015
qScript™ XLT One-Step RT-qPCR ToughMix®, Low ROX™	QuantaBio	Cat#95134
truChIP® Chromatin Shearing Kit	Covaris	Cat#PN 520154
Phusion Site-Directed Mutagenesis Kit	Thermo Scientific	Cat#F-541
Luciferase Reporter Gene Assay	Sigma-Aldrich	Cat#11814036001
Thermo Scientific™ Pierce™ Firefly Luciferase Glow Assay Kit	Fisher Scientific	Cat#PI16177
β-Gal Reporter Gene Assay, chemiluminescent	Sigma-Aldrich	Cat#11758241001
Dojindo Molecular Technologies, Inc. Cell Counting Kit-8 (Cell Proliferation Assay and Cytotoxicity Assay)	Fisher Scientific	Cat#NC0814108

**Deposited data**		

RNA-seq raw data files	This paper; Mendeley Data	http://doi.org/10.17632/v84nnrjw55.1; https://data.mendeley.com/datasets/v84nnrjw55/draft?a=3ef4815f-f1df-4f57-ae74-0d49e9388159
ChIP-seq raw data files	NCBI Gene Expression Omnibus	GEO: GSE205920
Existing, publicly available RNA-seq raw data files from study by Sablina et al. Functional genomics of Non-NF2 meningioma development and progression.	NCBI Gene Expression Omnibus	GEO: GSE156211

**Experimental models: Cell lines**		

293 human embryonic kidney cell line	Sigma-Aldrich	Cat#85120602
A549 human lung carcinoma cell line	Sigma-Aldrich	Cat#86012804
HBL-52 meningioma, benign cells	CLS Cell Lines Service GmbH	CLS Cat#300188/p692_HBL-52; RRID: CVCL_4220

**Oligonucleotides**		

TaqMan assay for *FGF3* RT-qPCR: FGF3-Fwd: TACCTGGCCATGAACAAGAG FGF3-Rev: CCGGGAGGCATACGTATTATAG FGF3-Probe: ATCCGCTCCACAAACTCGCACTC	This paper	N/A
TaqMan assay for *TRH* RT-qPCR: TRH-Fwd: GATCCCGGACCCATCCT TRH-Rev: GGTCAGGTTCAGGGTCAAAG TRH-Probe: TTGGTTGCTGCTCGCTCTGG	This paper	N/A
TaqMan assay for *KLF4* RT-qPCR: KLF4-Fwd: ATCCTTCCTGCCCGATCA KLF4-Rev: CTCTGGCATGCAGGAACC KLF4-Probe: ATGAGCTCTTGGTAATGGAGCGGC	This paper	N/A
TaqMan assay for *GAPDH* RT-qPCR: GAPDH-Fwd: GGTGTGAACCATGAGAAGTATGA GAPDH-Rev: GAGTCCTTCCACGATACCAAAG GAPDH-Probe: AGATCATCAGCAATGCCTCCTGCA	This paper	N/A

**Recombinant DNA**		

pGL3-Basic vector	Promega™	Cat#E1751
FGF3-Luc-3000	This paper	N/A
FGF3-Luc-2000	This paper	N/A
FGF3-Luc-1000	This paper	N/A
FGF3-Luc-540	This paper	N/A
FGF3-Luc-220	This paper	N/A
FGF3-Luc-177	This paper	N/A
-220-FGF3-Luc-IN2.1	This paper	N/A
-220-FGF3-Luc-IN2.2	This paper	N/A
-220-FGF3-Luc-52 kb	This paper	N/A
-220-FGF3-Luc-KCNK9	This paper	N/A
TRH-Luc-3000	This paper	N/A
TRH-Luc-2000	This paper	N/A
TRH-Luc-1000	This paper	N/A
TRH-Luc-402	This paper	N/A
TRH-Luc-235	This paper	N/A
hKLF4 cDNA in pDONR221	Harvard-DFCI plasmid repository	https://plasmid.med.harvard.edu/PLASMID
p3XFLAG-CMV™-7.1 Expression Vector	Sigma-Aldrich	Cat#E7533
p3XFLAG-KLF4	This paper	N/A
p3XFLAG-KLF4^K409Q^	This paper	N/A
p3XFLAG-KLF4 DBD	This paper	N/A
p3XFLAG-KLF4^K409Q^ DBD	This paper	N/A
pMAL-c6T Vector	New England BioLabs	Cat#N0378S
pMAL-KLF4 DBD	This paper	N/A
pMAL-KLF4^K409Q^ DBD	This paper	N/A

**Software and algorithms**		

GraphPad Prism 9	GraphPad Software	Prism - GraphPad
IGV 2.8.12	(Robinson et al., 2011; Thorvaldsdottir et al., 2013)	Home | Integrative Genomics Viewer (broadinstitute.org)
FastQC	(Andrews et al., 2010)	FastQC (illumina.com)
*BBduk*	(Bushnell, 2021)	BBTools - DOE Joint Genome Institute
BWA-mem	(Li and Durbin, 2009)	bwa.1 (sourceforge.net)
ENCODE blacklist	(Amemiya et al., 2019)	hg19-blacklist-README.pdf (broadinstitute.org)
MACS2	(Zhang et al., 2008)	MACS2 · PyPI
DiffBind	(Cairns et al., 2011)	Bioconductor - DiffBind
MEME-ChIP	(Machanick and Bailey, 2011)	MEME-ChIP - MEME Suite (meme-suite.org)
biomaRt	(Durinck et al., 2009)	Bioconductor - biomaRt
DESeq2	(Love et al., 2014)	Bioconductor - DESeq2
STAR	(Dobin et al., 2013)	GitHub - alexdobin/STAR: RNA-seq aligner

### Resource availability

#### Lead contact

Further information and requests for reagents should be directed to and will be fulfilled by the lead contact, Ian F. Dunn (ian-dunn@ouhsc.edu).

#### Materials availability

Plasmids and recombinant proteins generated in this study are available from the lead contact upon request.

### Experimental model and subject details

#### Cell lines

All cell lines were cultured in 37°C incubator with a humidified atmosphere of 5% CO_2_ in air. Human embryonic kidney cell line 293 (HEK293) (Sigma-Aldrich, 85120602) was cultured in Gibco™ EMEM (Fisher Scientific, 67-008-6) supplemented with 10% FCS, 2 mM glutamine, and 1% non-essential amino acids. Human lung carcinoma cell line A549 (Sigma-Aldrich, 86012804) was grown in Advanced DMEM (Fisher Scientific, 12491015) supplemented with 10% FCS and 2 mM glutamine. Both cell lines are adherent and were passaged as needed using TrypLE™ Express Enzyme (Fisher Scientific, 12-605-010). Benign meningioma HBL-52 cells were cultured in DMEM and Ham’s F-12, 50/50 Mix (Fisher Scientific, MT10092CV) supplemented with 2% FBS, 2 mM glutamine, 15 mM HEPES, 1.25 mg/mL BSA (Fraction V), 1xMEM NEAA, 1xMEM Vitamin Solution, 0.00535 mg/mL linoleic acid, 0.00625 mg/mL insulin, 0.00625 mg/mL transferrin, and 6.25 ng/mL selenium. HBL-52 is an adherent cell line with extremely slow growth. Medium was changed weekly and passaging was done as needed using TrypLE™ Express Enzyme.

#### Meningioma tissue

Tumor tissue from meningiomas was collected at the University of Oklahoma Medical Center with Institutional Review Board approval. Fresh tissue was excised and immediately submerged in RNAlater® Solution (Fisher Scientific, AM7023) and stored according to the manufacturer’s instructions.

### Method details

#### Plasmids and transfections

To clone KLF4 cDNA into expression vector, an ORF clone (HsCD00040749) was purchased from the Harvard-DFCI plasmid repository (https://plasmid.med.harvard.edu/PLASMID). The coding region of human KLF4 cDNA was amplified from the ORF clone and inserted into the p3XFLAG-CMV™-7.1 expression vector (Sigma-Aldrich, E7533) using EcoRV-BamHI restriction sites. Two variants of cDNA were cloned, KLF4 full length coding 479 aa long protein (aa 1–479) and KLF4 DBD coding 105 aa (aa 375–479). A Phusion Site-Directed Mutagenesis Kit (Thermo Scientific, F-541) was used to introduce a point mutation (T>G) for K409Q amino acid change in both constructs. All created constructs were confirmed by sequencing.

Transient transfections were carried out using Invitrogen™ Lipofectamine™ LTX Reagent with PLUS™ Reagent (Fisher Scientific, 15-338-100) according to the manufacturer’s instructions. Not more than 2 μg of total plasmid DNA per 10^6^ cells were used.

#### Western blot analysis

HEK293 cells were transfected with p3XFLAG-CMV™-7.1 expression vector expressing KLF4 or KLF4^K409Q^ proteins. Whole cell extracts were prepared by using 4x Laemmli Sample Buffer (BIO-RAD, 1610747) and incubation at 85°C for 10 min. Samples were run on an 8–16% Mini-PROTEAN® TGX™ Protein Gel (BIO-RAD, 4561105), transferred to nitrocellulose membrane, and probed with ANTI-FLAG® M2-Peroxidase (HRP) monoclonal antibody (Sigma, A8592). Other rabbit polyclonal antibodies used as primary were: anti-KLF4 (Sigma, SAB1300678); anti-KLF4 raised against c-terminus region, including K409 (Sigma, SAB2107958); and anti-Sp1 (Sigma, 07–645). Goat anti-rabbit IgG, HPR-linked (Cell Signaling Technologies, 7074) was used as secondary antibody. Sp1 levels were measured as loading controls.

#### Reporter gene assay

Promoter fragments of the human *FGF3* and *TRH* genes were cloned into pGL3-Basic vector (Promega) before the luciferase gene. Mini- and microsatellites DNA fragments were cloned immediately after the luciferase gene in construct containing minimal FGF3 promoter (−236 - +84 bp from TSS). All transfections into HEK293 cells were done using Invitrogen™ Lipofectamine™ LTX Reagent with PLUS™ Reagent (15-338-030, Fisher Scientific) according to the product’s instructions. Luciferase reporter constructs were co-transfected with p3XFLAG-CMV™-7.1 vector (E7533, Sigma-Ardrich) expressing human KLF4 or KLF4^K409Q^ proteins. Luciferase activity was measured 48 h post-transfections using Thermo Scientific™ Pierce™ Firefly Luciferase Glow Assay Kit (PI16177, Fisher Scientific) or Luciferase Reporter Gene Assay (11814036001, Sigma-Aldrich). Transfection efficiencies were normalized on co-transfected pSV-β-Galactosidase Control Vector (E1081, Promega). β-Gal levels were measured using β-Gal Reporter Gene Assay (11758241001, Sigma-Aldrich). Both reporters were quantified on the Cytation5 plate reader (BioTek). pGL3-Basic plasmid without the promoter served as a negative (no promoter) control. Its activity was set as 1 and promoter activities in all samples were calculated as fold increase over the “no promoter” sample. Error bars represent StDev from at least four independent experiments.

#### Electrophoretic mobility shift assay (EMSA)

Oligonucleotides (sense and antisense) spanning KLF4 binding sequences were [γ-^32^P]-ATP end-labeled, annealed, and incubated with nuclear extracts or recombinant KLF4 proteins in a binding buffer solution (10 mM Tris-HCl pH 7.5, 53 mM NaCl, 1 mM DTT, 0.01% Nonidet-P40, 5% glycerol, and Protein Inhibitors Cocktail) at RT for 30 min. In super-shift experiments, nuclear extracts were pre-incubated with 2 μg of anti-FLAG M2 (F3165, Sigma-Aldrich), anti-KLF4, or other antibody (as labeled in figures) for 30 min before adding the labeled probe (5’-GTGGCCTGGGCGGGACTGGG-3’). Protein-DNA complexes were separated by electrophoresis on a 5% polyacrylamide gel and exposed overnight to X-ray film. Sequences of oligonucleotides used as probes are shown in the figures.

#### DNase I footprinting assay

To purify KLF4 and KLF4^K409Q^ recombinant proteins for footprinting assay, we cloned the coding region of C-terminal 105 aa coding DBD of KLF4 and KLF4^K409Q^ into pMAL-c6T vector (New England Biolabs, N0378S) and expressed it in the BL21 bacterial strain. The recombinant proteins were purified using amylose magnetic beads (New England Biolabs, E8035S). DNase I footprinting of FGF3 promoter was performed as follows: a 360-bp fragment (from -276 to +84 bp relative to the TSS) of the *FGF3* promoter region was labeled with [γ-^32^P]-ATP at one end using T4 polynucleotide kinase and incubated with increasing amounts of recombinant protein (110 ng, 330 ng, 1 μg, 3 μg) at room temperature. After a 30-min incubation, the samples were digested with 0.3 units of DNase I for exactly 1 min before quenching enzyme activity with stop solution (0.05 mM EDTA, 0.125% SDS, 0.2M sodium acetate, and 0.1 mg/mL tRNA). Samples were purified by phenol chloroform extraction and ethanol precipitation. G + A ladder was prepared by treating labeled DNA fragment with 4% formic acid for 30 min at 37°C, followed by treatment with 1M piperidine for 30 min at 90°C, and precipitating with n-butanol. DNA fragments were resolved on an 8% denaturing polyacrylamide gel and exposed overnight to X-ray film. Footprinting analyses of other DNA fragments, i.e., *TRH* promoter, upstream *FGF3* promoter regions, *FGF3* intronic, and upstream microsatellites, were processed as described for *FGF3* promoter.

#### RNA extraction

For RNA-seq experiments, RNA was extracted using Direct-zol™ RNA MiniPrep Plus kit (Zymo Research Corporation, R2071). For all other experiments, cultured cells were lysed and total RNA was extracted with the RNeasy Plus mini kit (QIAGEN, 74136) with QIAshredder (QIAGEN, 79656) according to the manufacturer’s instructions. For biopsy tissues, samples were collected in Invitrogen™ RNAlater™ Stabilization Solution (Fisher Scientific, AM7023) and stored frozen at −80°C long term before RNA extraction.

#### Quantitative PCR (RT-qPCR)

Total cell RNA was used to measure gene mRNA levels by real-time qPCR. Reverse transcription and cDNA amplification were performed in one tube using qScript™ XLT One-Step RT-qPCR ToughMix®, Low ROX™ (VWR Quanta Biosciences™, 95134) on an Applied Biosystems 7500 Fast Real-Time PCR System (Fisher Scientific). Sample reactions were run in 3-6 replicates. Each mRNA analysis was run in a DuPlex PCR reaction with GAPDH as an internal control. Standard curves for each gene were run to verify the linear range of amplification. Input RNA was kept under 200 ng per reaction to stay within the linear range for GAPDH levels. Copy numbers for all genes of interest were calculated per 10^3^ or 10^6^ copies of GAPDH. All data were analyzed in Excel with the built-in analysis methods.

#### RNA-seq and differential expression (DE) analysis

HEK293 cells were transfected with p3XFLAG-CMV™-7.1 vector (E7533, Sigma-Aldrich) expressing human KLF4 or KLF4^K409Q^ proteins. Mock- and empty vector-transfected cells were used as negative controls. Then, 28–30 h post-transfection, cells were harvested and total RNA was purified using the Direct-zol™ RNA Miniprep Plus Kit (R2071, ZYMO Research) and subjected to full transcriptome sequencing. Three biological repeats were done for each condition. 3’-end RNA libraries were made using the Lexogen QuantSeq 3’ mRNA-Seq Library Prep Kit FWD for Illumina. Sequencing was done from Single-end 75 bp on an Illumina NextSeq High Output.

Post-sequence reads were quality-filtered for length and contaminants and were trimmed for Illumina adapters using BBDuk (Bushnell, 2021). Resulting reads were pseudo-aligned to coding regions of the human reference genome (GRCh38) using STAR (Dobin et al., 2013). Gene annotation was performed via the R package biomaRt (Durinck et al., 2009). Differential expression was calculated using the Wald test implemented in the R package DESeq2 (Love et al., 2014). Significantly differentially expressed genes were defined as those that had both an absolute log2 fold change ≥1 as well as a false discovery rate (FDR) adjusted p value ≤ 0.05 for each comparison independently.

#### ChIP-seq

Chromatin immunoprecipitation followed by sequencing (ChIP-seq) was performed using HEK293 cells transfected with p3XFLAG-CMV™-7.1 vector (E7533, Sigma-Ardrich) expressing human KLF4 or KLF4^K409Q^ proteins in 10-cm tissue culture dishes. Six independent biological replicates for each ChIP-seq condition were analyzed. The truChIP® Chromatin Shearing Kit (PN520154, Covaris) was used for preparing samples for high-throughput sequencing. All steps were carried out according to the kit procedure. Briefly, 24 h post-transfection, cells were fixed with formaldehyde and lysed to prepare nuclei. Their chromatin was shared for 16 min/sample in a 1-mL volume using an E220 Evolution sonicator (Covaris) with the instrument settings described in the kit (above). Chromatin concentration was measured on NanoDrop and diluted 1:1 in Covaris 2x IP Dilution Buffer. Diluted chromatin was pre-cleared by centrifugation (10,000x g for 5 min at 4°C) and supernatants were used for immunoprecipitation (IP). Five hundred μg of chromatin (the equivalent of 100 μg of pure genomic DNA content) for each sample was immunoprecipitated with 40 microliters of anti-FLAG® M2 Magnetic Beads (M8823, Sigma-Aldrich) in a rotary holder overnight at 4°C. The beads were washed once with 1x RIPA Lysis Buffer (20–188, Sigma-Aldrich), followed by four more washes with RIPA Wash buffer (RIPA Lysis buffer with an increased concentration of NaCl to 500 mM). The washed beads were reverse-crosslinked by treating with Proteinase K overnight at 65°C in a 200-μL volume. Precipitated genomic DNA was purified using a GeneJET PCR Purification Kit (FERK0701, Fisher Scientific).

#### Sequencing alignment and peak calling

ChIP-seq libraries were prepared according to Swift Bioscience protocols and were sequenced using 150 bp paired-end sequencing on an Illumina NovaSeq, producing an average of 177,095,140 reads per library.

ChIP-seq reads were examined for technical artifacts with FastQC (Andrews et al., 2010). No aberrant technical behavior was identified. Reads were trimmed for adapter sequences and decontaminated for sequencing artifacts by BBDuk (Bushnell, 2021). Trimming options were set to ktrim = right trimming, mink = 11, hdist = 1, qin = 33, and tpe and tbo options enabled. BBDuk’s list of Illumina sequencing adapters was used to perform adapter trimming. Decontamination was done against PhiX adapters and BBDuk’s database of sequencing artifacts. Decontaminated reads were aligned to version GRCh38 of the human reference genome using BWA-mem (Li and Durbin, 2009) with default options. Aligned reads were filtered to ensure a minimum of one mapped read per pair, a minimum Phred quality score of 10, and exclusion from the ENCODE genomic region blacklist (Amemiya et al., 2019). After all filtering steps, samples averaged 83% read retention with Relative Strand Cross-correlation (RSC) values between 1.3 and 1.6, demonstrating strong signal enrichment. Peaks were called with respect to the input chromatin library using MACS2 (Zhang et al., 2008) with the following options: *-g hs -q 0.05 -f BAMPE –keep-dup all.* Samples averaged a fraction of reads in peaks (FRiP) between 4% and 6%. The peaks were visualized in Integrative Genomic Viewer (Robinson et al., 2011; Thorvaldsdottir et al., 2013).

#### Consensus binding site motif analysis

Quality-filtered ChIP-seq alignments and MACS2 called peaks were input into the DiffBind (Cairns et al., 2011) R package and used to develop condition-specific consensus peak sites appearing in the KLF4- and KLF4^K409Q^-transfected cell lines. A consensus peak appeared minimally in 3 out of 6 replicates per condition. The MEME-ChIP software suite (Machanick and Bailey, 2011) was used to perform *de novo* motif analysis, scanning for the top 30 most enriched motifs with width between 9 and 11 bp. Input FASTA sequences were constructed from 200 bp windows around consensus peak centers.

#### Cell proliferation assay

HBL-52 cells were seeded in 96-well plates at 1,000 cells per well in 100 μL of complete medium containing 2% FBS and all cell line-specific supplements as described in the experimental model and subject details section. Recombinant human FGF3 (R&D Systems, 1206-F3) was added to medium up to a final concentration of 0.1 μg/mL in the presence of 1 μg/mL of heparin (StemCell Technologies, 07980). Recombinant human FGF1 (R&D Systems, 232-FA) was used at a final concentration of 0.1 μg/mL in the presence of 10 μg/mL of heparin. Recombinant human EGF (R&D Systems, 236-EG) was used at final concentration of 5 ng/mL. Cell proliferation was measured at days 7, 12, and 18 for FGF3 stimulation, and at days 8 and 19 for FGF1 and EGF stimulations. Since HBL-52 cells grow extremely slow and require intricate growing conditions, we used a cell proliferation and cytotoxicity assay (Dojindo Molecular Technologies, Inc., Cell Counting Kit-8 [CCK-8]; Fisher Scientific, NC0814108). It is a sensitive, non-toxic colorimetric assay for the determination of the number of viable cells in cell proliferation and cytotoxicity assays. It correlates well with [^3^H]-thymidine incorporation assays and can be substituted for these assays. Ten μL of the CCK-8 solution were added directly to each well of the 96-well plate on designated days post-stimulation, incubated 4 h in the CO_2_ incubator, and the absorbance at 450 nm was measured on the Cytation5 plate reader (BioTek). After the measurements, wells with cells were washed with PBS and replenished with fresh medium with the same stimulators and let continuously grow further in the tissue culture incubator. Further measurements were carried out in the same way. Cell proliferation was measured in eight technical replicates for each condition and repeated three times. All data were analyzed in Excel with the built-in analysis methods. Data are represented as the mean ± StDev.

### Quantification and statistical analysis

All reporter gene assays and cell proliferation assays data were analyzed and plotted using Excel 2016 with the built-in analysis methods and presented as the mean ± StDev. Gene expression levels by RT-qPCR for all genes of interest were calculated by comparative ΔC_T_ experiment runs on AB7500 Fast machine and analyzed using the 7500 Software v2.3. The ΔC_T_ data were recalculated for each gene mRNA copies per 1 thousand (10^3^) or 1 million (10^6^) copies of GAPDH in Excel. In RNA-seq DE analysis, differential expression was calculated using the Wald test implemented in the R package DESeq2 (Love et al., 2014). Significantly differentially expressed genes were defined as those that had both an absolute log2 fold change ≥1 as well as a false discovery rate (FDR) adjusted p value ≤ 0.05 for each comparison independently.

## Data Availability

• RNA-seq raw data files have been deposited on Mendeley. ChIP-seq raw and processed data files have been deposited on NCBI Gene Expression Omnibus. The DOI and GEO accession numbers are listed in the key resources table. • This paper does not report original code. • Any additional information required to reanalyze the data reported in this paper is available from the lead contact upon request.

## Supporting Citations

The following references appear in the Supplemental information: Goujon et al. (2010); Heger and Holm (2000); Schwartz et al. (2000).

## Supplemental information

Document S1. Figures S1–S7 and Table S1

Table S2. ChIP-seq in HEK293 cells: KLF4-binding statistics used for genome-wide enhancer motif search, related to Figure 4

Table S3. ChIP-seq in HEK293 cells: Top 10% of variable signal annotations context, related to Figure 4

Table S4. RNA-seq in HMCs: Activated and repressed DEGs in three different comparisons as labeled on the tab of each page, related to Figure 7

Table S5. RNA-seq in HMCs: DEGs in three different comparisons as labeled on the tab of each page, related to Figure 7
