# Supplementary material for: Mutated KLF4(K409Q) in meningioma binds STRs and activates FGF3 gene expression
Source: iScience. 2022 Aug 3;25(8):104839. doi: 10.1016/j.isci.2022.104839 (PMC9391581; doi:10.1016/j.isci.2022.104839)
Supplement: Document S1. Figures S1–S7 and Table S1 [file mmc1.pdf]

iScience, Volume *25*

Supplemental information

Mutated KLF4(K409Q) in meningioma binds STRs and activates *FGF3* gene expression

Alla V. Tsytsykova, Graham Wiley, Chuang Li, Richard C. Pelikan, Lori Garman, Francis A. Acquah, Blaine H.M. Mooers, Erdyni N. Tsitsikov, and Ian F. Dunn

HEK293

Gene	$KLF4^{K409Q}$/KLF4 Fold change
FGF3	39.29
CALML5	37.04
OSGIN1	31.01
COL17A1	14.62
UCKL1-AS1	10.97
GJB1	10.09
DEGS2	8.93
SLC38A8	6.63
IGSF21	5.65
KCNK9	5.62
TMEM233	4.33
RRAD	3.55
ASCL2	3.17
SLIT1	3.15
COL11A2	3.08
CAMK2B	3.02
NOVA2	3.02
CFAP65	2.93
SGCA	2.85
MUC2	2.75
MARVELD3	2.57
TMC6	2.55
MYOD1	2.5
TMEM52	2.4
RIMS4	2.34
MFSD2A	2.25
GPSM1	2.08
ERBB3	2.07
KCNC1	2.06
CLIC3	0.37
SLC17A7	0.35
GABRD	0.25
PLIN4	0.21
KRT17	0.19
ALPG	0.05
TRH	0.03

A549

Gene	$KLF4^{K409Q}$/KLF4 Fold change
CALML5	26.17
FGF3	24.75
IGSF21	19.05
MYOD1	13.49
SLC38A8	12.68
SLIT1	11.93
KCNK9	10.67
SGCA	8.22
GJB1	7.25
NOVA2	6.87
UCKL1-AS1	6.84
COL11A2	6.42
KCNC1	6.25
DEGS2	5.44
TMEM233	4.56
MUC2	4.22
CAMK2B	4.04
MARVELD3	3.93
RIMS4	3.72
RRAD	3.34
GPSM1	2.88
TMC6	2.81
COL17A1	2.77
TMEM52	2.68
ERBB3	2.68
ASCL2	2.59
CFAP65	2.54
OSGIN1	2.27
MFSD2A	2.22
CLIC3	0.5
SLC17A7	0.41
PLIN4	0.29
KRT17	0.24
GABRD	0.21
TRH	0.08
ALPG	0.01

Figure S1. Differential gene expression analysis of RNA-seq results in HEK294 and A549 cell lines with over-expressed WT or mutant KLF4 proteins.

Differentially expressed genes (DEGs) found in both HEK293 and A549 cells are sorted by $KLF4^{K409Q}$/KLF4 fold induction from highest to lowest in each cell line. Top two $KLF4^{K409Q}$-dependent genes are highlighted in orange and top two KLF4-dependent genes are highlighted in blue. Related to Figure 2.

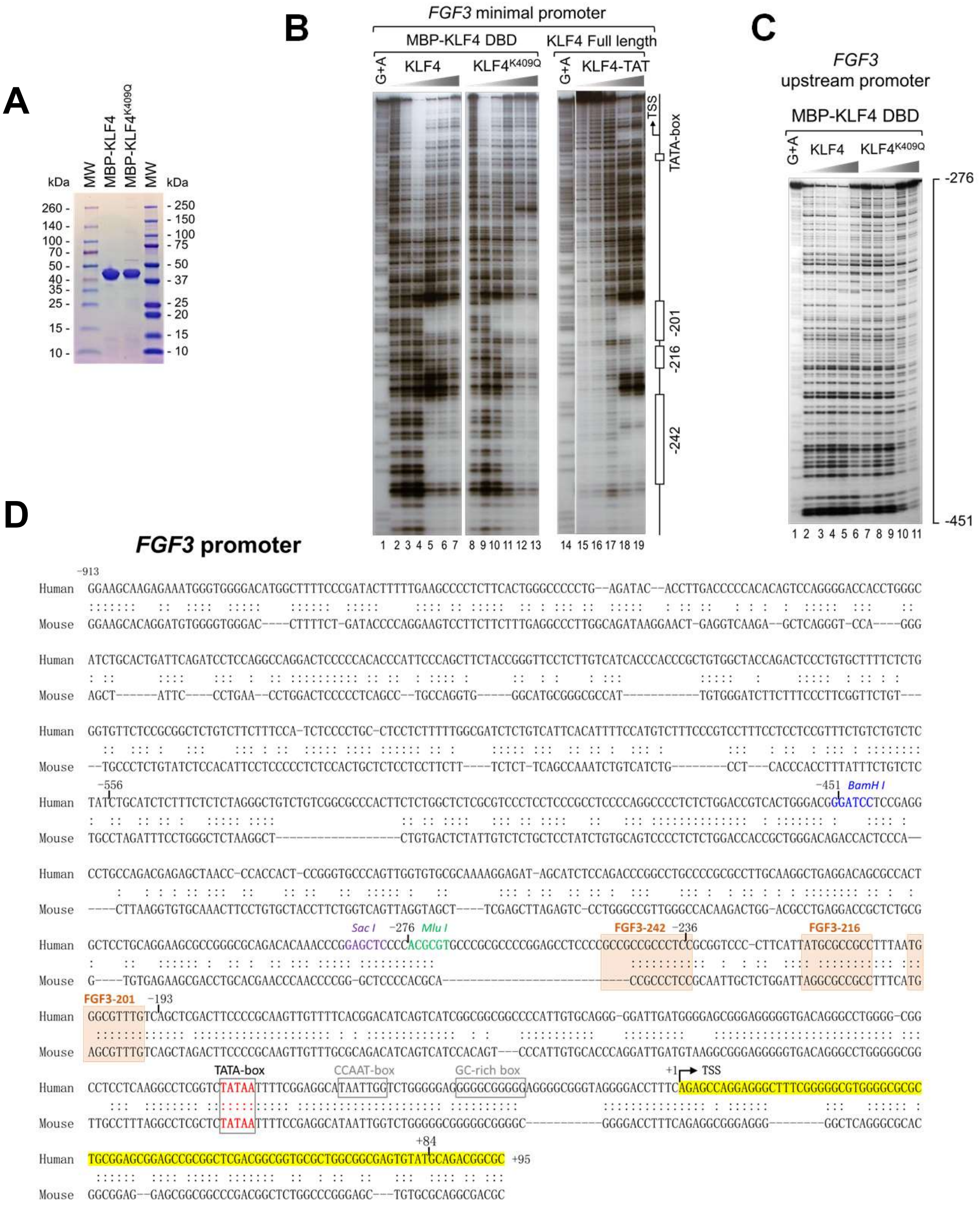

Figure S2. Strong binding of KLF4^{K409Q} to several sites in the *FGF3* minimal promoter region.

(**A**) Expression of MBP-KLF4 and MBP-KLF4^{K409Q} DBD proteins in BL21 *E. coli*. SDS gel of *E. coli* lysates. Each lane contains ~3 ug of recombinant protein. MW: molecular weight standards. (**B**) MBP-KLF4 DBD and full-length KLF4-TAT fusion proteins display identical protection of DNA from DNase I digestion. Quantitative DNase I footprinting analysis of the *FGF3* minimal promoter using increasing concentrations of recombinant proteins as indicated. (**C**) Negligible KLF4 binding to the region upstream of the *FGF3* minimal promoter. Footprinting analysis of the *FGF3* upstream promoter from -276 to -451 bp relative to the TSS as described in (B). (**D**) Sequence alignment of human and mouse *FGF3* promoter regions. First *FGF3* exon is highlighted in yellow. TSS is marked by the arrow. Brown boxes with names indicate KLF4^{K409Q} binding regions. Positions of truncated promoter fragments used in reporter gene assays are labeled as (-556, -236, -193, +84). Restriction enzymes used in footprinting experiments are labelled by blue (*BamH I*) and green (*Mlu I*) fonts. Restriction enzyme *Sac I* (purple font) and regulatory elements (CCAAT and GC-rich boxes) outlined by grey rectangles were used in promoter studies in (Galdemard et al., 2000). Related to Figure 3.

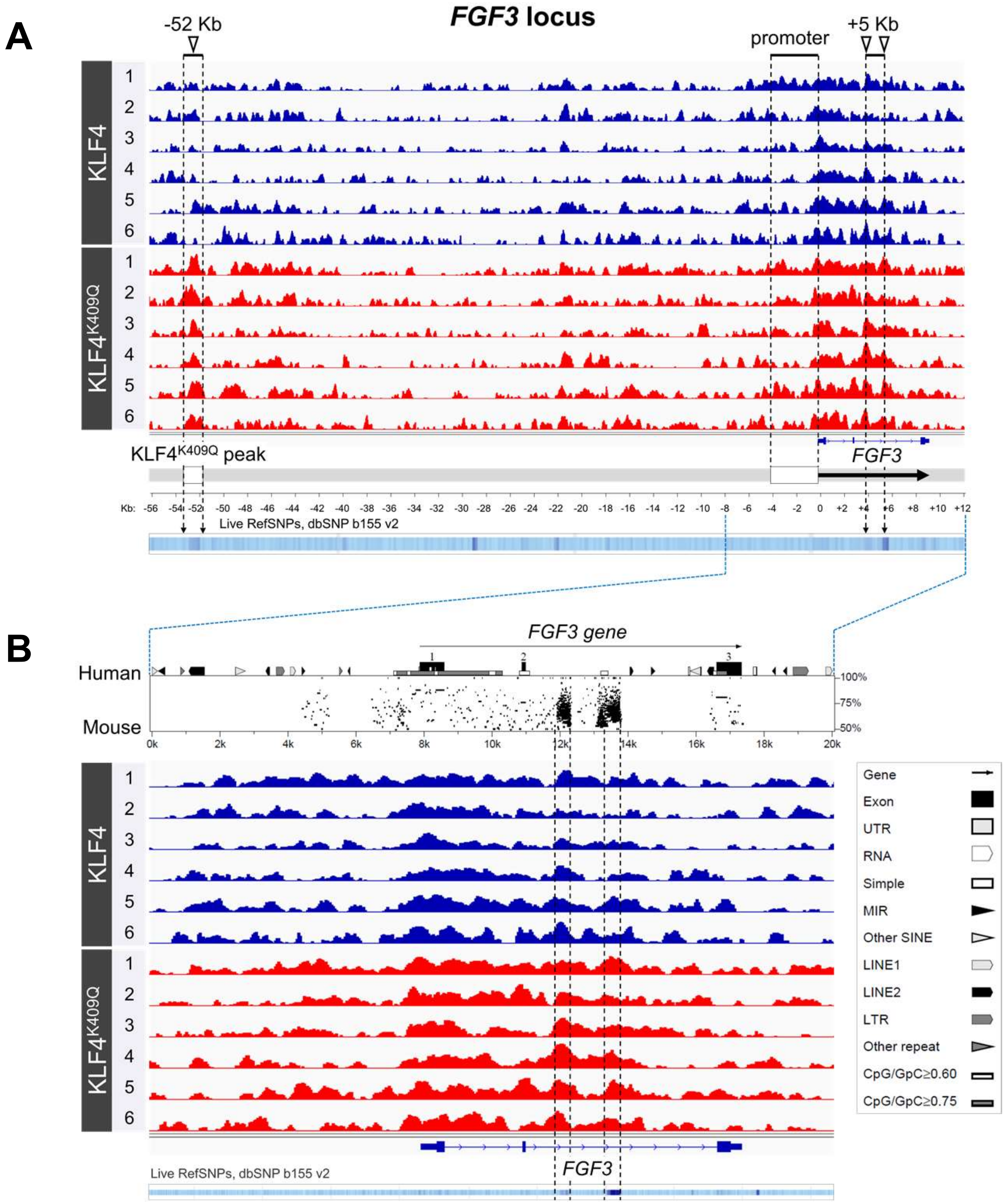

Figure S3. ChIP-seq analysis of the *FGF3* locus in HEK293 cells with KLF4 or KLF4^{K409Q} over-expression.

(**A**) Bigwig tracks, KLF4 (blue) and KLF4^{K409Q} (red), display log_2 ratio of KLF4 ChIP-seq coverage relative to input. Six independent biological replicates for each ChIP-seq condition were analyzed. Schematic position and direction of *FGF3* gene transcription are shown below the plots. Alignment with heatmap of RefSNPs is also shown (bottom track). Position of promoter and short tandem repeat (STR) DNA regions are depicted above the plots. (**B**) Zoom in on *FGF3* gene and its close locus surroundings. Alignment of genomic sequence conservation analysis between human and mouse *FGF3* loci by PipMaker (Schwartz et al., 2000) (top track) with ChIP-seq bigwig tracks for the human *FGF3* locus (middle tracks) and a heatmap of RefSNPs (bottom track). The percent identity (*y-axis*) plot (Pip) of human (*x-axis*) to mouse sequence is lined up with KLF4-binding peaks in STRs IN2.1 and IN2.2 in *FGF3* intron 2 and regions of high SNPs accumulation in RefSNPs track below. Six independent biological replicates for each ChIP-seq condition (KLF4 and KLF4^{K409Q}) are labeled on the left side. Related to Figure 5A.

A

Region	Length (bp)	DNA Strand	Percent Composition						
			A	C	G	T	GC	Purine	Pyrimidine
FGF3 promoter	320	coding	13	24	42	21	67	55	45
FGF3-52 kb	1,278	coding	17	13	44	26	58	61	39
FGF3-IN2.1	457	template	22	3	50	25	54	72	28
FGF3-IN2.2	492	template	20	2	53	26	55	73	28

B

STR	Indices	Period Size	Copy Number	Consensus Size	Percent Matches	Percent Indels	Alignment Score	Percent Composition			
								A	C	G	T
FGF3-52 kb	1--964	19	50.8	19	83	1	989	14	12	47	24
	878--1010	57	2.3	57	78	0	131	16	9	48	24
	1074--1112	19	2.1	19	90	0	60	25	7	38	28
FGF3-IN2.1	68--145	12	6.5	12	74	11	61	14	0	60	25
	60--408	8	43.3	8	79	7	339	19	0	55	24
FGF3-IN2.2	27--458	8	54	8	86	0	504	17	1	55	25
	27--455	24	17.9	24	88	2	585	17	1	55	25

C

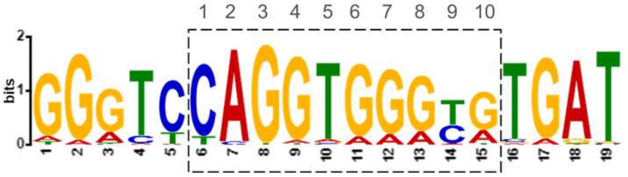

FGF3-52 kb
19 bp repeats

D

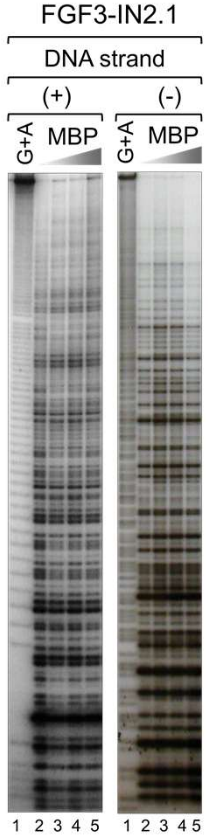

Figure S4. DNA composition analysis of KLF4^{K409Q}-binding short tandem repeats (STRs) in the *FGF3* locus.

(**A**) Analysis of *FGF3* minimal promoter and three STR sequences shows GATC count as percentage of each nucleotide. (**B**) *FGF3* locus STR sequence analysis details using a tandem repeat finder tool (Benson, 1999). (**C**) Consensus motif derived from 60 x 19 bp repeats from FGF3-52 kb STR using MEME software (Bailey and Elkan, 1994). Sequence letter height is correlated with conservation. Nucleotides resembling a newly identified KLF4^{K409Q}-binding motif in Figure 4F are numbered and framed in a rectangle. (**D**) Recombinant mannose binding protein (MBP) purified from BL21 *E. coli* does not bind DNA. Negative control for MBP non-specific binding to DNA. Quantitative DNase I footprinting analysis of the 463 bp DNA fragment from FGF3-IN2.1 STR was performed with increasing amounts of MBP, as indicated. Both sense (+) and antisense (-) DNA strands (as marked and identified by G+A ladder) were labelled and tested. No non-specific binding was observed. Related to Figures 5B and 6C.

A

sample	condition	batch	file
GFP_1	GFP	B1	GSM4726806_GFP.Gene.Count.txt.gz
GFP_2	GFP	B2	GSM4726807_GFP_1.Gene.Count.txt.gz
GFP_3	GFP	B3	GSM4726808_GFP_4.Gene.Count.txt.gz
GFP_4	GFP	B4	GSM4726809_GFP_6.Gene.Count.txt.gz
WT_KLF4_1	WT_KLF4	B1	GSM4726810_WT_KLF4.Gene.Count.txt.gz
WT_KLF4_2	WT_KLF4	B2	GSM4726811_KLF4_WT_1.Gene.Count.txt.gz
WT_KLF4_3	WT_KLF4	B3	GSM4726812_KLF4_WT_4.Gene.Count.txt.gz
WT_KLF4_4	WT_KLF4	B4	GSM4726813_KLF4_WT_6.Gene.Count.txt.gz
KLF4_K409Q_1	KLF4_K409Q	B1	GSM4726814_K409Q_KLF4.Gene.Count.txt.gz
KLF4_K409Q_2	KLF4_K409Q	B2	GSM4726815_KLF4_409_1.Gene.Count.txt.gz
KLF4_K409Q_3	KLF4_K409Q	B3	GSM4726816_KLF4_409_4.Gene.Count.txt.gz
KLF4_K409Q_4	KLF4_K409Q	B4	GSM4726817_KLF4_409_6.Gene.Count.txt.gz

B

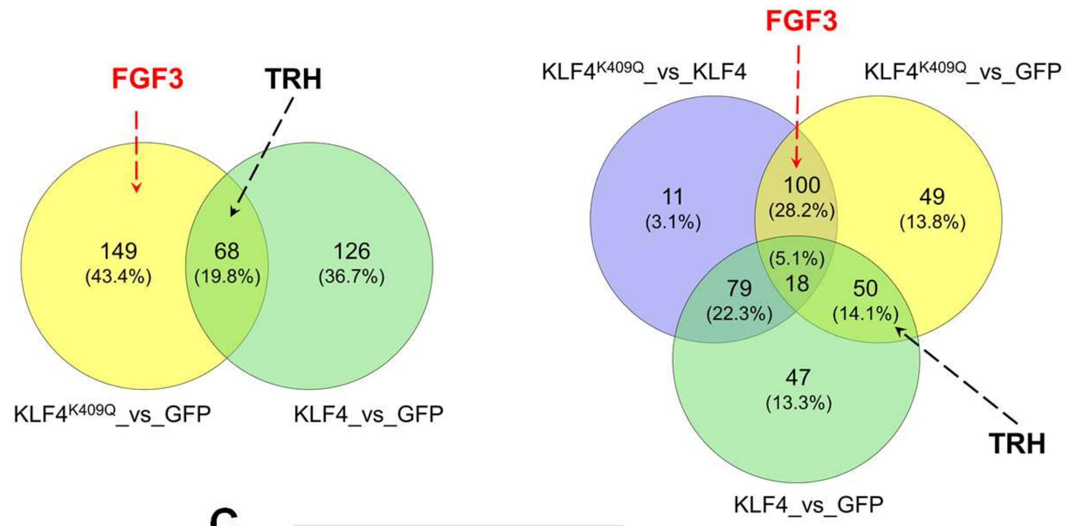

C

Gene	**KLF4^{K409Q}/KLF4 Fold change**
FGF3	134.03
MYOD1	112.32
SGCA	71.72
SLIT1	39.73
DEGS2	35.54
MUC2	24.65
COL11A2	19.74
CALML5	16.49
SLC38A8	16.24
NOVA2	10.87
RIMS4	10.82
CAMK2B	10.78
ASCL2	7.95
MARVELD3	5.9
KCNC1	5.4

Figure S5. RNA-seq analysis of primary human meningeal cells (HMCs) transduced with viral vectors expressing GFP alone or fusion proteins with WT or mutant KLF4.

(**A**) The table depicts the samples and raw data files from NCBI Gene Expression Omnibus (GEO: GSE156211) used in meta-analysis. Three conditions with four biological replicates were analyzed. (**B**) RNA-seq meta-analysis of raw data files described in (A). Venn diagram showing the overlap of significant DEGs in HMCs transduced with viral vectors expressing GFP alone or fusion proteins with KLF4 and KLF4^{K409Q} (left panel). Right panel displays the same two sets of DEGs as in the left panel with an additional set of significant DEGs from KLF4^{K409Q} vs. WT KLF4 pair analysis. The gene sets, where *FGF3* and *TRH* were found, are indicated by red and black arrows, respectively. (**C**) List of DEGs found by RNA-seq (meta-analysis) in primary HMCs shared with DEGs found in both HEK293 and A549 cells. Top two KLF4^{K409Q}–dependent genes in cell lines, *FGF3* and *CALML5*, are highlighted in orange. Related to Figure 7.

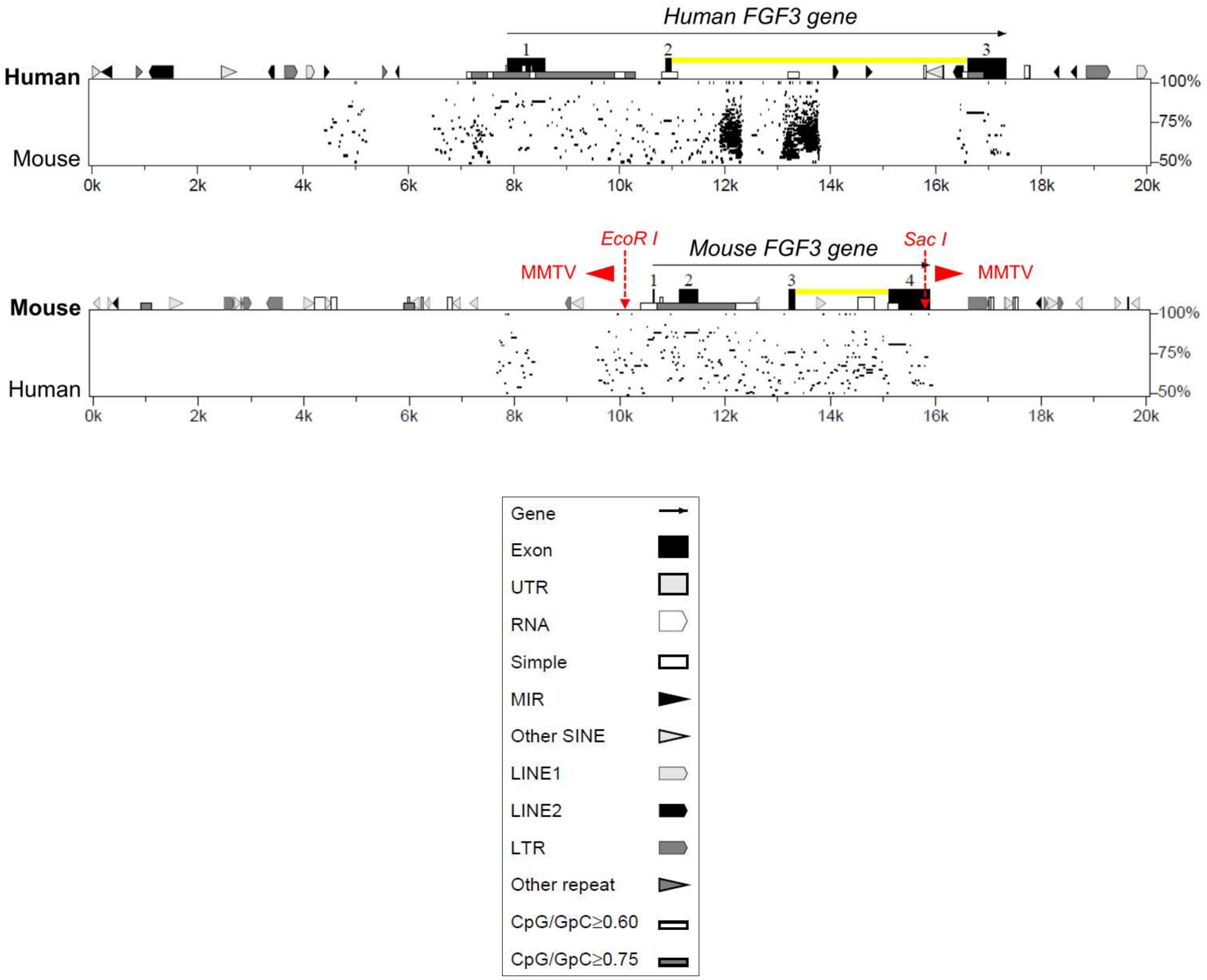

Figure S6. Mouse *FGF3* contains no intronic STRs.

PipMaker alignment of *FGF3* loci: Human vs. Mouse (top plot) and Mouse vs. Human (bottom plot). Mouse intron 3, which corresponds to intron 2 in humans (highlighted by yellow line), is almost 5 kb shorter than human intron 2 and lacks STRs. Previously published location and orientation of MMTV proviruses relative to mouse *FGF3* (*int-2* transcription unit) are indicated by red arrows (Dickson et al., 1984). Related to Figure 5A.

```
< IN2.1 (457 bp)
AGACACAGCTTCCAGGACACTGACTGATGAATGGACTGAATGTCAGATGGATAGGTGTATGGATGGATGGGTGAGT
GGGTGGGTGGATGGATGGGTGAGTGGGTGGGTGGATAGGTGGATGGGTGAGTGGATGAGTGGATGGGTGGATAGGT
GGATGGATGGATGGATGGATGGATGGATAGGTGGATGGGTGGATGGGTGGATGGGTAAATGGGTGGATGGGTGGAT
GGGTGGATGGATGGATGGATAGGTGGATGGTGGATGGGTAAATGGAAGGATGGGTGGATGGGTGGATGGATGGATG
GATAGGTGGATGGATGGATGGGTGGATGGGTGGATGAGTGGATGGATGGATGGGTGGATGGGTGGATGAGTGGATG
GGTGAATGGGTGGATGGGTGGATGGGTGAGTGGATGGGAGGAAGGAGGGAGTAGCCTCTGGACCAGAGAAAATGAA
G
# 5'3' Frame 1
RHSFQDTD--MD-MSDG-VYGWMGEWVGGWMGEWVGG-VDG-VDEWMGG-VDGWMDGWMDRWMGGWVDG-
MGGWVDGWMDGWIGGWWMGKWKDGWMGGWMDG-
VDGWMGGWVDEWMDGWVDGWMSGWVNGWMGGWVSGWEEGGSSLWTRENE
------------------------------------------------------------------------
No. of Repeats|Total Score|Length  |Diagonal| BW-From|   BW-To|   Level
             2|      94.95|      20|      22|       1|      20|       1
------------------------------------------------------------------------
   1-   20 (49.05/11.12)  VDGWMGGWVDEWMDGWVDGW
  25-   44 (45.90/10.02)  VNGWMGGWVSGWEEGGSSLW
------------------------------------------------------------------------
# 5'3' Frame 2
DGTASRTLTDEWTECQMDRCMDGWVSGWVDGWVSGWVDRWMGEWMSGWVDRWMDGWMDGWIGGWVDGWMGKWVDGW
MGGWMD-VDGGWVNGRMGGWVDGWMDRWMDGWVDGWMSGWMDGWMGG-VDG-MGGWVDG-VDGRKEGVASGPEKMK
------------------------------------------------------------------------
No. of Repeats|Total Score|Length  |Diagonal| BW-From|   BW-To|   Level
             2|     103.19|      20|      22|      39|      60|       1
------------------------------------------------------------------------
  39-   58 (53.23/10.99) WMGEWMSGWVDRWMDGWMDG
  63-   82 (49.96/ 7.31) WVDGWMGKWVDGWMGGWMDG
------------------------------------------------------------------------
------------------------------------------------------------------------
No. of Repeats|Total Score|Length  |Diagonal| BW-From|   BW-To|   Level
             2|      42.98|      10|      22|       9|      18|       2
------------------------------------------------------------------------
   9-   18 (21.55/ 8.39)  DEWTECQMDR
  29-   38 (21.43/ 8.30)  DGWVSGWVDR
------------------------------------------------------------------------
# 5'3' Frame 3
TQLPGH-LMNGLNVRWIGVWMDG-VGGWMDG-VGGWIGGWVSG-VDGWIGGWMDGWMDG-
VDGWMGGWVNGWMGGWVDGWMDRWMVDG-MEGWVDGWMDGWIGGWMDGWMGG-
VDGWMGGWVDEWMGEWVDGWMGEWMGGRRE-PLDQRK-

                             No repeats found
# 3'5' Frame 1
LHFLWSRGYSLLPPIHSPIHPSTHSPIHSSTHPPIHPSTHPPIHPSIHPPIHPSIHPSTHPSIYPSTIHLSIHPST
HPPIHPFTHPPIHPSTYPSIHPSIHPPIHPSTHPLTHPPIHPPTHPSIHPPTHPSIHTPIHLTFSPFISQCPGSCV
------------------------------------------------------------------------
No. of Repeats|Total Score|Length  |Diagonal| BW-From|   BW-To|   Level
             3|     166.94|      26|      26|      73|      98|       1
------------------------------------------------------------------------
  38-   63 (50.78/ 8.03) STHPPIHPSIHPPIHPsiHPSTHPSI......
  83-  110 (60.68/11.39) FTHPPIHPSTYPSIHPsiHPPIHPSTH....P
 111-  142 (55.47/ 9.62) LTHPPIHPPTHPSIHPptHPSIHTPIHltfsP
------------------------------------------------------------------------
------------------------------------------------------------------------
No. of Repeats|Total Score|Length  |Diagonal| BW-From|   BW-To|   Level
             2|      80.28|      18|      42|      20|      37|       2
------------------------------------------------------------------------
  20-   37 (42.49/12.31)  HPST.HSPIHSSTHPPIHP
  64-   82 (37.80/ 9.97)  YPSTiHLSIHPSTHPPIHP
------------------------------------------------------------------------
# 3'5' Frame 2
FIFSGPEATPSFLPSTHPSTHPPIHPSTHPPIHPSIHPLIHPSTHPSIHLSIHPSTHPPILPFTHPPSTYPSIHPP
IHPSTHLPIHPSTHPPIHPSIHPSIHLSTHPLIHSPIHLSTHPLTHPSTHPLTHPSIHLSI-HSVHSSVSVLEAVS
------------------------------------------------------------------------
No. of Repeats|Total Score|Length  |Diagonal| BW-From|   BW-To|   Level
             3|     151.79|      22|      22|      18|      39|       1
------------------------------------------------------------------------
  18-   38 (53.14/ 9.52)  PSTHPPIHPSTHPP.IHPSIHP.
  55-   76 (42.06/ 6.06)  .STHPPILPFTHPPsTYPSIHPP
  79-  100 (56.59/10.60)  PSTHLPIHPSTHPP.IHPSIHPS
------------------------------------------------------------------------
------------------------------------------------------------------------
No. of Repeats|Total Score|Length  |Diagonal| BW-From|   BW-To|   Level
             2|      51.71|      12|      18|     105|     116|       4
------------------------------------------------------------------------
 105-  116 (25.79/ 7.94)  THPLIHSPIHLS
 125-  136 (25.91/ 8.01)  THPLTHPSIHLS
------------------------------------------------------------------------
# 3'5' Frame 3
SFSLVQRLLPPSSHPLTHPPIHPFTHPLIHPSTHPSIHSSTHPPIHPSTYPSIHPPIHPSFHLPIHHPPIHPSIHP
STHPPIYPSTHPPIHLSIHPSIHPSTYPPIHSSTHPSTYPPTHSPIHPPTHSPIHPYTYPSDIQSIHQSVSWKLC
------------------------------------------------------------------------
No. of Repeats|Total Score|Length  |Diagonal| BW-From|   BW-To|   Level
             3|     193.40|      33|      34|      18|      51|       1
------------------------------------------------------------------------
  11-   41 (60.04/ 7.95)  .....PSSHPltHPPIHP.FTHPLIHPSTHPSIHSST
  42-   78 (66.78/11.92)  HPPIhPSTYPsiHPPIHPsFHLPIHHPPIHPSIHPST
  79-  110 (66.58/ 9.86)  HPPI....YPstHPPIHL.SIHPSIHPSTYPPIHSST
------------------------------------------------------------------------
------------------------------------------------------------------------
No. of Repeats|Total Score|Length  |Diagonal| BW-From|   BW-To|   Level
             2|      57.13|      13|      18|     111|     123|       2
------------------------------------------------------------------------
 111-  123 (29.29/11.72)  HPSTYPPTHSPIH
 131-  143 (27.85/10.68)  HPYTYPSDIQSIH
------------------------------------------------------------------------
```

```
< IN2.2 (492 bp)
ACTGGTGAACGAAGGACTGAATGTCAGATGGGTGGGTGGATGGATGGATGGATGGGTGGATGGATGGATGGATAGA
TGGGTGGGTGGATGGATGGATGGATGGGTGGATGGATGGATGGATGGATGGGTGGATGGGTGGATGGATGGATGGG
TGGATGGCTGGATGGATGGATGGGTGGATGGATGGATAGGTGGATGGATGGATAGGTGGATGGATGGATGGGTGGA
TGGCTGGATGGATGGATGGGTGGATGGGTGGATGGATGGATGGATGGATGGATGGATGGATGGATGGGTGGATGGG
TGGATGGGTGGATGGGTGGATGGCTGGTTGGATGGATGGGTGGATGGATGGATAGGTGGATGGATGGATGGGTGGA
TGGCTGGATGGATGGGTGGATTGCTGGATGGATTGGTGGATGGGTTGATGGGTGGATGGGTGGATGGGTGGATGGG
TGAAAGGATGGAAGGAAGGAGGGAGCATGGACGGAT
# 5'3' Frame 1
TGERRTECQMGGWMDGWMGGWMDG-MGGWMDGWMGGWMDGWMGGWVDGWMGGWLDGWMGGWMDRWMDG-
VDGWMGGWLDGWMGGWVDGWMDGWMDGWMGGWVDGWMGGWLVGWMGGWMDRWMDGWVDGWMDGWIAGWIGGWVDGW
MGGWVDG-KDGRKEGAWTD
------------------------------------------------------------------------
No. of Repeats|Total Score|Length  |Diagonal| BW-From|   BW-To|   Level
             2|     169.28|      30|      30|       3|      32|       1
------------------------------------------------------------------------
   3-   32 (85.92/10.55)  GWMGGWLDGWMGGWVDGWMDGWMDGWMGGW
  35-   64 (83.36/10.05)  GWMGGWLVGWMGGWMDRWMDGWVDGWMDGW
------------------------------------------------------------------------
# 5'3' Frame 2
LVNEGLNVRWVGGWMDGWVDGWMDRWVGGWMDGWVDGWMDGWVDGWMDGWVDGWMDGWVDGWIGGWMDRWMDGWVD
GWMDGWVDGWMDGWMDGWMDGWVDGWMGGWVDGWLDGWVDGWIGGWMDGWMAGWMGGLLDGLVDGLMGGWVDGWMG
ERMEGRREHGR
------------------------------------------------------------------------
No. of Repeats|Total Score|Length  |Diagonal| BW-From|   BW-To|   Level
             4|     319.60|      30|      30|      33|      62|       1
------------------------------------------------------------------------
  33-   62 (87.27/12.80)  GWVDGWMDGWVDGWMDGWVDGWMDGWVDGW
  65-   94 (88.28/13.03)  GWMDRWMDGWVDGWMDGWVDGWMDGWMDGW
  97-  126 (83.35/11.90)  GWVDGWMGGWVDGWLDGWVDGWIGGWMDGW
 129-  157 (60.70/ 6.70)  GWMGGLLDGLVDGLMGGWVDGWMGERMEG.
------------------------------------------------------------------------
------------------------------------------------------------------------
No. of Repeats|Total Score|Length  |Diagonal| BW-From|   BW-To|   Level
             2|      63.32|      13|      14|       4|      16|       2
------------------------------------------------------------------------
   4-   16 (30.10/ 7.30)  EGLNVRWVGGWMD
  20-   32 (33.22/ 8.85)  DGWMDRWVGGWMD
------------------------------------------------------------------------
# 5'3' Frame 3
W-TKD-MSDGWVDGWMDGWMDGWIDGWVDGWMDGWMDGWMDGWMGGWMDGWMAGWMDGWMDG-
VDGWIGGWMDGWMAGWMDGWMGGWMDGWMDGWMDGWMGGWVDGWMAGWMDGWMDG-
VDGWMGGWLDGWVDCWMDWWMG-WVDGWMGGWVKGWKEGGSMDG
------------------------------------------------------------------------
No. of Repeats|Total Score|Length  |Diagonal| BW-From|   BW-To|   Level
             2|      93.80|      17|      22|      20|      36|       2
------------------------------------------------------------------------
  20-   36 (46.74/ 8.03)  GWVDGWMDGWMDGWMDG
  40-   56 (47.06/ 8.12)  GWMDGWMAGWMDGWMDG
------------------------------------------------------------------------
# 3'5' Frame 1
IRPCSLLPSILSPIHPSTHPPINPSTNPSSNPPIHPAIHPSIHPPIHPSTHPSNQPSTHPPIHPSTHPSIHPSIHP
SIHPSTHPSIQPSTHPSIHLSIHPPIHPSTHPSIQPSTHPSIHPSTHPSIHPSTHPSIHPPTHLSIHPSTHPSIHP
PTHLTFSPSFTS
------------------------------------------------------------------------
No. of Repeats|Total Score|Length  |Diagonal| BW-From|   BW-To|   Level
             4|     214.55|      22|      22|      74|      95|       1
------------------------------------------------------------------------
  34-   55 (52.35/ 9.96)  IHPAIHPSIHPPIHPSTHPSNQ
  75-   95 (50.66/ 9.38)  .HPSIHPSTHPSIQPSTHPSIH
  98-  119 (56.73/11.45)  IHPPIHPSTHPSIQPSTHPSIH
 122-  143 (54.80/10.79)  THPSIHPSTHPSIHPPTHLSIH
------------------------------------------------------------------------
# 3'5' Frame 2
SVHAPSFLPSFHPSTHPPIHPSTHPPIHPAIHPSIQPSTHPSIHLSIHPPIHPTSHPPIHPSTHPPIHPSIHPSIH
PSTHPPIHPSSHPPIHPSTYPSIHLSIHPPIHPSSHPPIHPSTHPPIHPSIHPPIHPSIHPPIYPSIHPPIHPSIH
PPI-HSVLRSP
------------------------------------------------------------------------
No. of Repeats|Total Score|Length  |Diagonal| BW-From|   BW-To|   Level
             2|      80.21|      14|      14|     125|     138|       1
------------------------------------------------------------------------
 125-  138 (40.11/ 6.28)  PSIHPPIHPSIHPP
 141-  154 (40.11/ 6.28)  PSIHPPIHPSIHPP
------------------------------------------------------------------------
------------------------------------------------------------------------
No. of Repeats|Total Score|Length  |Diagonal| BW-From|   BW-To|   Level
             4|     226.84|      22|      22|      73|      94|       2
------------------------------------------------------------------------
   9-   30 (58.43/10.17)  PSFHPSTHPPIHPSTHPPIHPA
  33-   54 (51.82/ 8.09)  PSIQPSTHPSIHLSIHPPIHPT
  73-   94 (59.99/10.66)  PSIHPSTHPPIHPSSHPPIHPS
  97-  118 (56.60/ 9.60)  PSIHLSIHPPIHPSSHPPIHPS
------------------------------------------------------------------------
# 3'5' Frame 3
PSMLPPSFHPFTHPPIHPSTHQPIHQSIQQSTHPSSHPPIHPSTYPSIHPSIQPAIHPSTHPPIHPSIHPSIHPSI
HPPIHPSIHPAIHPSIHPPIHPSTYPSIHPSIHPAIHPSIHPPIHPSIHPSIHPSIHPSTHPSIHPSIHPSIHPST
HPSDIQSFVHQ
------------------------------------------------------------------------
No. of Repeats|Total Score|Length  |Diagonal| BW-From|   BW-To|   Level
             6|     233.80|      14|      14|      57|      70|       1
------------------------------------------------------------------------
  41-   54 (36.09/ 6.10)  HPSTYPSIHPSIQP
  57-   70 (40.92/ 7.83)  HPSTHPPIHPSIHP
  73-   86 (39.63/ 7.36)  HPSIHPPIHPSIHP
  97-  110 (38.05/ 6.80)  HPSTYPSIHPSIHP
 113-  126 (39.63/ 7.36)  HPSIHPPIHPSIHP
 133-  146 (39.49/ 7.31)  HPSTHPSIHPSIHP
------------------------------------------------------------------------
```

Figure S7. Hypothetical protein translation analysis of STRs in *FGF3* intron 2 (IN2.1 and IN2.2).

Sequence of hypothetical peptides by using ExPASy translation tool (ExPASy - Translate tool) and RADAR software (Goujon et al., 2010; Heger and Holm, 2000) for detection and alignment of repeats in protein sequences. Both STRs were translated in both directions and peptide repeats are shown in color letters based on identity/homology of amino acids. Where more than two peptides were identified in one reading frame, the longest one (underlined) was analyzed for repeats. Related to Figure 5B.

Clinical characteristics of primary tumors

								per 10^6 GAPDH	per 10^3 GAPDH
Sample ID	WHO grade	Histologic Type	Patient age	Sex	Mutated genes	TRAF7 mutation	KLF4 mutation	FGF3 mRNA copies	KLF4 mRNA copies
M-014	I	Transitional	51	F	NF2, CREBZF, JAK2, KDM6B				405
M-015	I	Meningothelial	57	F	NF2, COL6A3				945
M-017	I	Meningothelial	59	M	MAP2K1, MSH6, TERF1, TRAP1				3215
M-018	I	Transitional	59	F	NF2, EGFR				389
M-019	I	Angiomatous	48	F	BCORL1				1019
M-020	I	Atypical/ Anaplastic	24	M	NF2, FGFR3				1330
M-023	I	Meningothelial	57	F	TERT, BCORL1, FGFR3, MSH6				300
M-024	I	Angiomatous	57	F	NF2, NOTCH1, KMT2D, QKI				332
M-026	I	Meningothelial	58	F	TRAF7, AKT1, TET2	G536S			1955
M-027	I	Meningothelial	55	F	TRAF7, AKT1	K615E			2133
M-028	I	Meningothelial	74	F	MSH6				550
M-029	I	Meningothelial	73	M	POLR2A, KMT2C				537
M-030	I	Secretory	51	F	TRAF7, KLF4, POT1	K387Q	K409Q	5.2	921
M-031	I	Meningothelial	53	F	NCR				3075
M-032	I	Secretory	68	M	TRAF7, KLF4, KMT2B	G536S	K409Q		1519
M-033	I	Meningothelial	59	F	BRAF, GLI2, KMT2C, PIK3R2, POLE				480
M-034	I	Meningothelial (psammomatous component)	54	F	TRAF7, AKT1, FGFR1, KMT2D, TET1	I368del			2913
M-035	I	Meningothelial	27	M	TRAF7, AKT1, KMT2C, SUFU, TSC2	L519R			3748
M-036	I	Transitional	74	M	NF2, KDM5A, KMT2C				241
M-037	I	Meningothelial	66	M	NF2, KLF4, KMT2B		G239_P255del		496
M-038	I	NR	46	F	TRAF7, APC	N520S			520
M-039	I	Meningothelial	74	M	TRAF7, AKT1	N632K			385
M-040	II	Atypical	65	F	TRAF7, AKT1, ARID1A, SETD2	G390E			750
M-041	I	Meningothelial	77	F	NF2, TERT				403
M-043	II	Atypical meningioma with chordoid component	51	F	TRAF7, GLI3, SETD2, TET2	Y577C			781
M-044	I	Meningothelial	67	F					1599
M-045	I	Spindle cell with sclerotic changes	54	M	RB1, GLI3				2125
M-047	I	Meningothelial (psammomatous component)	63	M	SMO, FGFR3, GLI2				283
M-048	I	Secretory	64	F	TRAF7, KLF4, GLI2	N520S	K409Q	21.4	1735
M-049	I	Meningothelial	47	f	TRAF7, IDH2	R641C, C130Y			424
M-051	I	Meningothelial	72	F	ARID2, NF2, NOTCH2, PTCH1				407
M-052	I	Meningothelial	89	F	TRAF7	N520S			689
M-053	I	Meningothelial	66	M	PRKAR1A, TET2				173
M-055	II	Atypical or anaplastic	79	M	NF2, TET2				264
M-056	I	Microsystic	24	M	CDKN2C, KDM6A				219
M-057	I	Meningothelial	45	F					104
M-058	I	Meningothelial	49	F	TRAF7, KMT2D, MYC	C388Y			225
M-059	I	Meningioma	53	F	PIK3CA, TERT, PTCH, SMO				120
M-060	I	Atypical meningioma with chordoid component	55	F					4392
M-061	I	Meningothelial	63	M	SMO, KMT2B, SUFU				246
M-062	I	Meningioma	55	F	NF2, ATM, WRN				155
M-063	I	Atypical meningioma with chordoid component	73	F	NF2, CDKN2A, ARID1A, SETD2				402
M-064	I	Meningioma	61	F	NF2, CHEK2, SMARCB1, ARID2, MSH3				500
M-065	I	Meningioma	72	F	TERT, FGFR1				322
M-066	I	Secretory	46	F	TRAF7, KLF4, TCF12	G536S	K409Q	83.3	3899
M-067	I	Meningothelial	56	F	TRAF7, PIK3CA, ATRX	N520S			4050
M-068	I	Meningothelial	61	F	TRAF7, KLF4, POT1, BRAF, GNA11, MLH1	R653P	K409Q	9.8	2542
M-069	I	Meningothelial	53	F	TRAF7, AKT1	Y538C			2468
M-070	I	Meningothelial	64	F	TRAF7, KLF4, KMT2D	S645R	K409Q	298.4	1712
M-071	II	Atypical meningioma	63	F	CHEK2, NF1, NF2, KMT2B, KMT2D, PTCH2				794

Table S1. List of meningioma tissue samples with identified mutations and their clinical characteristics.

Tumors with K409Q mutation in KLF4 are highlighted in orange. Related to STAR Methods.
